# Dissecting the Gene Network of Dietary Restriction to Identify Evolutionarily Conserved Pathways and New Functional Genes

**DOI:** 10.1371/journal.pgen.1002834

**Published:** 2012-08-09

**Authors:** Daniel Wuttke, Richard Connor, Chintan Vora, Thomas Craig, Yang Li, Shona Wood, Olga Vasieva, Robert Shmookler Reis, Fusheng Tang, João Pedro de Magalhães

**Affiliations:** 1 Integrative Genomics of Ageing Group, Institute of Integrative Biology, University of Liverpool, Liverpool, United Kingdom; 2 Department of Biology, University of Arkansas, Little Rock, Arkansas, United States of America; 3 Institute of Integrative Biology, University of Liverpool, Liverpool, United Kingdom; 4 Central Arkansas Veterans Healthcare System, University of Arkansas for Medical Sciences, Little Rock, Arkansas, United States of America; 5 Department of Geriatrics and Department of Biochemistry and Molecular Biology, University of Arkansas for Medical Sciences, Little Rock, Arkansas, United States of America; Stanford University Medical Center, United States of America

## Abstract

Dietary restriction (DR), limiting nutrient intake from diet without causing malnutrition, delays the aging process and extends lifespan in multiple organisms. The conserved life-extending effect of DR suggests the involvement of fundamental mechanisms, although these remain a subject of debate. To help decipher the life-extending mechanisms of DR, we first compiled a list of genes that if genetically altered disrupt or prevent the life-extending effects of DR. We called these DR–essential genes and identified more than 100 in model organisms such as yeast, worms, flies, and mice. In order for other researchers to benefit from this first curated list of genes essential for DR, we established an online database called GenDR (http://genomics.senescence.info/diet/). To dissect the interactions of DR–essential genes and discover the underlying lifespan-extending mechanisms, we then used a variety of network and systems biology approaches to analyze the gene network of DR. We show that DR–essential genes are more conserved at the molecular level and have more molecular interactions than expected by chance. Furthermore, we employed a guilt-by-association method to predict novel DR–essential genes. In budding yeast, we predicted nine genes related to vacuolar functions; we show experimentally that mutations deleting eight of those genes prevent the life-extending effects of DR. Three of these mutants (*OPT2*, *FRE6*, and *RCR2*) had extended lifespan under *ad libitum*, indicating that the lack of further longevity under DR is not caused by a general compromise of fitness. These results demonstrate how network analyses of DR using GenDR can be used to make phenotypically relevant predictions. Moreover, gene-regulatory circuits reveal that the DR–induced transcriptional signature in yeast involves nutrient-sensing, stress responses and meiotic transcription factors. Finally, comparing the influence of gene expression changes during DR on the interactomes of multiple organisms led us to suggest that DR commonly suppresses translation, while stimulating an ancient reproduction-related process.

## Author Summary

Dietary restriction has been shown to extend lifespan in diverse, evolutionarily distant species, yet its underlying mechanisms remain unknown. We first constructed a database of genes essential for the life-extending effects of dietary restriction in various model organisms and then studied their interactions using a variety of network and systems biology approaches. This enabled us to predict novel genes related to dietary restriction, which we validated experimentally in yeast. By comparing large-scale data compilations (interactomes and transcriptomes) from multiple organisms, we were able to condense this -*omics* information to the most conserved essential elements, eliminating species-specific adaptive responses. These results lead us to the rather surprising conclusion that lifespan extension by a restricted diet commonly may exploit an ancient rejuvenation process derived from gametogenesis.

## Introduction

Dietary restriction (DR) in defined factors (such as calories or specific amino acids) without causing malnutrition delays the aging process, protects against age-related diseases (e.g. metabolic and cardiovascular disease, neurodegeneration and cancer) and extends lifespan in evolutionarily distant species, from unicellular yeast to rodents [1], [2]. Furthermore, there is evidence that DR might delay the aging process in non-human primates [3] and perhaps in humans as well [4]. While multiple studies in model organisms have shown that DR extends lifespan, the underlying molecular mechanisms remain largely unknown [2], [5], [6].

The mechanisms by which DR retards aging have been shown in model organisms to be mediated by genetic pathways, many of which are evolutionarily conserved and operate in humans. Considerable evidence indicates that DR is mediated by discrete signaling events that elicit specific genetic programs [1], [2], [7], [8]. Mutations in a number of genes, in fact, have been shown to prevent, shift or disrupt the lifespan-extending effect of DR, in many cases without altering the lifespan under *ad libitum* (AL) conditions. Such genes, which we call DR-essential, might specifically interfere with these signaling cascades and the program which leads to lifespan extension under DR. Understanding the interactions of such genes in a systematic way may provide important new clues regarding DR-mediated life-extension mechanisms.

In the post-genomic era, the vast amount of -*omics* data provides an opportunity to understand biological processes in a systematic fashion via data integration, network construction and analyses. Networks in biology exist on various levels, derived from the interactions between genes, transcripts, proteins and metabolites, and extending to the interactions between cells, tissues, organs and even between organisms. Examples at the molecular level are protein-protein interactions, gene-regulatory and metabolic networks. Biological networks tend to have certain discrete topological properties: They are usually scale-free (i.e. only a few nodes, termed “hubs”, have many connections, while the majority of nodes have relatively few connections), modularly composed and hierarchically structured. Scale-free networks are robust against random perturbation, but at the same time are sensitive to targeted disruption of the hubs [9]. The modular composition is due to the presence of groups of highly interconnected nodes (i.e. clusters) which perform certain biological activities (for instance protein complexes or pathways) with relatively sparse external connectivity. Lastly, these network modules associate with one another in a hierarchical manner. The challenge, however, is to integrate and combine the various networks in order to decipher biological function and gain new insights into the process under study.

Aging-associated genes are more conserved at the molecular level [10] and have a significantly higher node degree (i.e. number of interactions in a network) than expected by chance. Human orthologs of aging-associated genes found in model organisms form a continuous network and almost all of the hubs are also implicated in several age-related diseases [11]. The networks of age-related disease genes and aging-associated genes significantly overlap and approximately half of the common genes participate in signal transduction. Additionally, general disease genes have more connections to aging genes than expected by chance [12]. Numerous network studies on aging have been conducted with fruitful results [13]–[15]. To date, however, network studies on DR have been limited, in part because of a lack of adequate datasets. Therefore, we constructed GenDR, a database of DR-related genes.

Since GenDR is the first database of DR-related genes, we wished to assess its utility by performing the first network-based dissection of DR using DR-essential genes. DR can induce numerous changes in organisms, and a variety of different DR regimens have been employed. Our rationale is that by concentrating only on genes essential for DR-induced life-extension we are able to narrow down the various DR-related processes to those affecting aging. We used our list of DR-essential genes to investigate their evolutionary and network properties and identify common regulators of DR-induced lifespan extension. Novel DR-essential genes were predicted from the network and tested experimentally in yeast, which revealed new genes crucial for DR. We then integrated diverse types of data and performed a variety of network and systems biology analyses to reveal common pathways and mediators of DR effects, including putative transcription factors. Our work demonstrates the use of network approaches to study DR mechanisms, and we make our list of DR-essential genes available online for other researchers to use (http://genomics.senescence.info/diet/).

## Results

### Construction of GenDR, a Database of DR–Related Genes

A list of DR-essential genes was compiled from the literature. A DR-essential gene was defined as one which when genetically manipulated in a given organism blocks or disrupts the life-extending effect of DR. This criterion applies even if it was shown for only a single DR regimen. Over 100 genes were identified, mostly from the traditional biomedical model organisms (Table 1). In mice, for example, the only DR-essential gene found so far is the growth hormone receptor (*Ghr*) gene of which homozygous knockout mutants were long-lived and DR failed to further extend their lifespan [16]. In the case of yeast, DR-essential genes were annotated separately for replicative and chronological lifespan. However, both were combined for the analyses presented below, in order to focus on mechanisms universal to DR. For each gene, its orthologs in other model organisms and in humans (if any) were retrieved from public databases (see Materials and Methods).

**Table 1:** Number of DR–essential genes and orthologs in GenDR.

Species	Genes	Genes+Orthologs
Fission yeast	6	54
Budding yeast	70	170
Nematode	42	137
Fruit fly	19	187
House mouse	1	218
Norway rat	0	217
Rhesus monkey	0	200
Human	0	226

Shown is the number of genes reported in different model organisms to be essential for DR (“Genes”) and number of DR-essential genes complemented with the orthologs of genes reported in other organisms (“Genes+Orthologs”).

We created a publically accessible database of DR-essential genes and their orthologs, called GenDR, which offers an important new tool to researchers working on the genetics of DR and of aging. Although other studies have emphasized the importance of genes that disrupt life-extending effects of DR [1], [2], [17], to our knowledge this is the first compilation of a list of such genes. The GenDR database employs the same system and interface as our widely-used GenAge database of aging-related genes [18]. Our inclusive selection criteria allow GenDR to incorporate a broad range of genes. Because information on each gene and reason(s) for its selection are included, however, researchers are able to focus on subsets of the database that are most relevant to their work. GenDR will be useful both as an informational website and as a research tool, and it is freely available online for the research community to use (http://genomics.senescence.info/diet/).

### GenDR Is Enriched in Conserved Longevity Genes and Pathways

Given that DR works across diverse model organisms, we expected that GenDR can guide the search for evolutionarily conserved longevity mechanisms. Therefore, we analyzed GenDR at three levels: the sequences of genes/proteins, the interactome, and enriched pathways.

**Figure 1:**
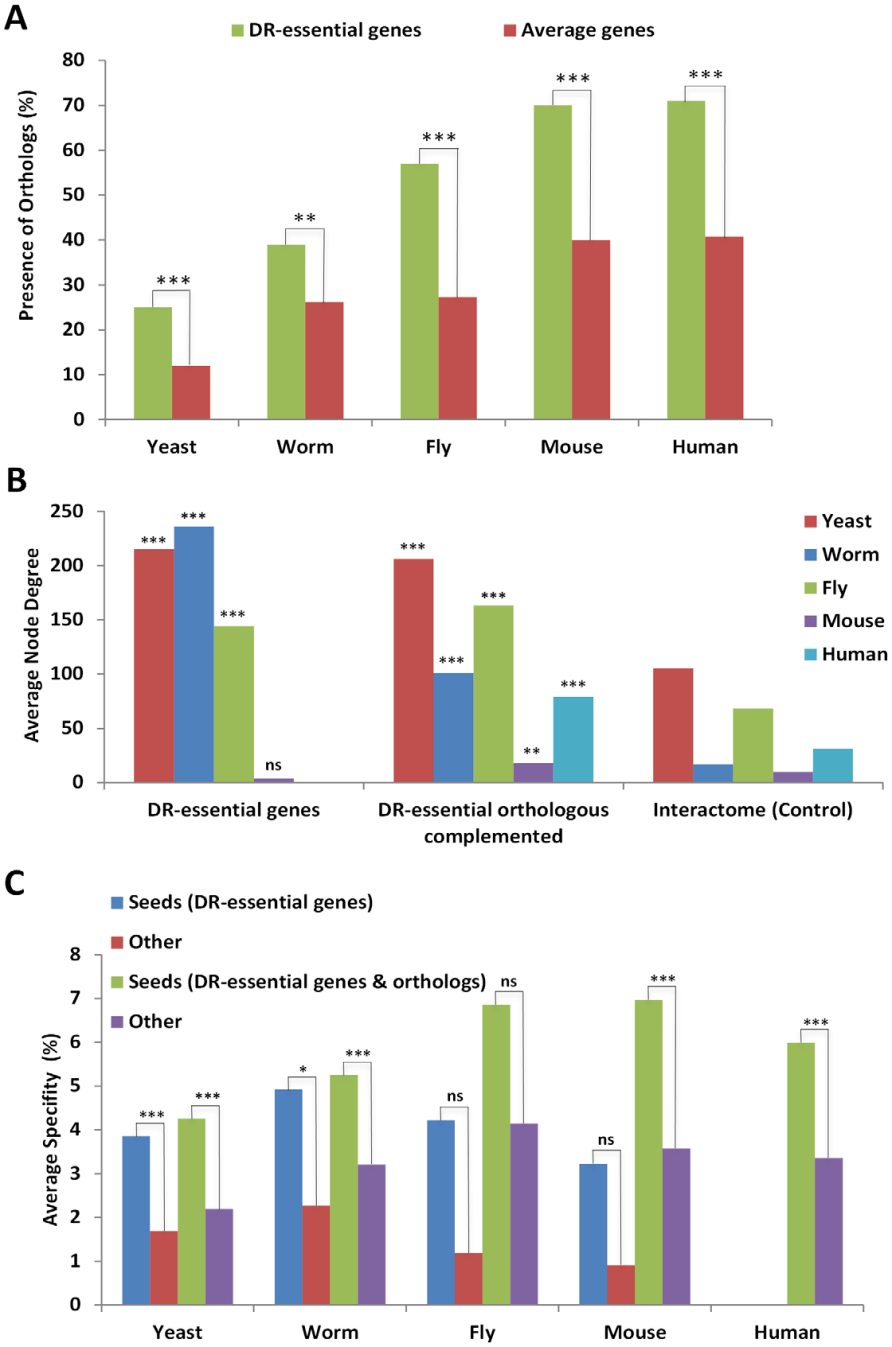
DR–essential genes are molecularly conserved and form a tight interaction network. A, The proportion of genes (shown as percentages) with orthologs from multiple species in the specified organisms is in all cases higher (by 10–30%) for DR-essential genes than expected by chance. B, The average degree (number of interactions) for DR-essential genes and DR-essential gene orthologs (the ortholog-complemented set) is higher than the interactome-wide average which serves as control. C, The average percentage of specificity in interconnectivity (100× specific interactions with seed genes/all interactions) of DR-essential genes to each other is higher than for other genes in interactomes, and complementation with orthologs of DR-essential genes from other species further increases the specificity of interconnectivity. ns, P>0.05; *, P<0.05; **, P<0.01; ***, P<0.001.

To test whether GenDR reflects the conserved effects of DR on longevity, we investigated the molecular evolution of genes essential for DR. In agreement with our hypothesis, the percentage of orthologs of DR-essential genes across all tested species is higher than expected by chance (Figure 1A). Likewise, the average dN/dS ratio, a measure of molecular evolution rate, as well as dN and dS rates, for DR-essential gene orthologs in mammalian species pairs were lower than expected by chance (Table S1 and Figure S1). As such, it appears that indeed DR is evolutionarily conserved at the genetic level.

Interaction networks of DR-essential genes plus their orthologs were created by integrating molecular interaction information from various sources: physical, genetic and other interactions such as regulatory relationships were retrieved from public databases (see Materials and Methods). The human orthologs of DR-essential genes form an interaction network in which the total interactions appear to follow approximately a power law distribution (Figure S2). This is consistent with a scale-free network in which only a few nodes (often called “hubs”) are highly connected. In other words, a few hubs contribute highly to the DR network connectivity, as has been observed in other biological networks [11], [12].

DR-essential genes as well as their orthologs have a higher node degree than expected by chance when compared to the whole interactome (Figure 1B). Thus, DR-essential genes tend to be located in the center of the interactome rather than in the periphery. As anticipated, DR-essential genes interact with each other more than expected by chance (Figure 1C). Adding orthologs to the DR-essential gene seed lists (orthology complementation) increased the specificity (interaction with seed genes/total interactions) of the connections between seed genes and enabled the generation of interaction networks for species with few or no known DR-essential genes, such as mammals. DR-essential as well as aging-related genes are significantly enriched for signaling genes (Table S2). Nonetheless, DR-essential genes are even more evolutionarily conserved than aging-related genes (Figure S3) and have a higher average degree than signaling genes (Figure S4), indicating that these properties (evolutionary conservation and high degree) are not secondary effects expected for subsets of aging-related genes and signaling genes, respectively.

One issue, however, is that the finding of higher conservation and interconnectivity for DR-essential genes compared to other genes might reflect a selection bias of researchers, who tend to study genes that have orthologs in multiple species and/or those in pathways associated with DR. Because node degree, presence of orthologs and sequence conservation are thought to be related, we investigated the correlations among these variables for human genes. We found that log(degree) and number of species with orthologs are positively correlated (Pearson correlation coefficient, *r* = 0.36; p<10^−200^), which implies that the higher the number of connections (i.e. interactions) of a human gene the higher the number of species which have orthologs of that gene. Similarly, log(degree) was weakly negatively correlated with log(dN/dS) (*r* = −0.08; p<10^−11^), indicating that genes with more connections have slightly lower dN/dS ratios and hence more conserved sequences. Finally, the log(ortholog number) was also found to be negatively correlated with log(dN/dS) (*r* = −0.24; p<10^−100^), suggesting that genes which have orthologs across many species also tend to have lower dN/dS ratios, as expected. An interpretation of these results is that genes with high degree (more connectivity) tend to also have high numbers of orthologs and low dN/dS ratios. This makes sense, since important hubs in the network have more constraints limiting their evolutionary divergence. Next, we performed a multiple linear regression analysis (with log-transformed data) to derive equations relating each of these three variables as a function of the other two. We used the equations to determine whether DR-essential gene orthologs have higher or lower values than predicted from the other two variables. The results reveal that DR-essential gene orthologs have an average degree much higher than expected (73 vs. 12), a slightly higher number of species with orthologs (10 vs. 9) and dN/dS ratios higher than the expected value (0.08 vs. 0.04). Thus, a high node degree appears to be the most critical feature of DR-essential gene orthologs.

To ascertain which processes and functions are common to the DR gene network, DR-essential genes plus their significant interactors (p<0.05 according to specificity of interaction with DR-essential genes, as described in the Materials and Methods) for each species were subjected to functional enrichment analysis using DAVID (Database for Annotation, Visualization and Integrated Discovery; Table S3A). Common to all DR-essential gene interaction networks in yeast, worm and fly were the terms *aging* (p<10^−27^) and *mitochondrion* (p<10^−10^). Terms common to ortholog-complemented networks in yeast, worms, flies, mice and humans were categories related primarily to *phosphorylation signaling*, among others (Table S3B). Kinase signaling and protein phosphorylation are also dramatically suppressed in very long-lived PI3K-null mutant worms [19], [20]. We then asked which genes among the significant interaction partners were common to multiple organisms. Although no homologous group was significant at False Discovery Rate (FDR)<0.05 (which appears to be too strict for comparisons across multiple species), by the more relaxed criterion of having a binomial p-value<0.05, four homologous groups were nominally significant: *CAB39*, the genes encoding 14-3-3 proteins, sirtuins, and *AHCY* encoding S-adenosylhomocysteinase.

### Guilt-by-Association Discovers Novel Vacuolar DR–Essential Gene Functions

To predict novel DR-essential candidate genes we used a guilt-by-association strategy [21]. Essentially, the logic behind this approach is that a gene with more interactions than expected by chance with genes associated with a given process (i.e. DR-mediated lifespan extension) is likely to also play a role in that process (see Materials and Methods). The candidate genes with highest significance in their connectivity to DR-essential genes were *YGR272C* (*EFG1*; Exit From G1) in *Saccharomyces cerevisiae*, *B0280.10* (*pot-1*; Protection of Telomeres 1 (Pot1) homolog) in *Caenorhabditis elegans*, and *Akt1* (serine-threonine kinase involved in insulin-like signaling) in *Drosophila melanogaster*. After orthologous complementation, among the highly significant candidate genes were, for example, *PAI3* (essential Proteinase A inhibitor) in *S. cerevisiae*, *hcf-1* (human host cell factor (HCF-1) homolog) in *C. elegans*, *CG7333* (an organic cation transmembrane transporter) in *D. melanogaster*, *Raf1* (MAP kinase kinase kinase) in *Mus musculus*, and *ACD* (involved in telomere maintenance and meiosis) in humans. In yeast, worm, and fruit fly, respectively, 372, 1317, and 202 genes were significant at a p-value<0.05. After orthologous complementation there were 264 genes significant in yeast, 228 in worm, 743 in fruit fly, 2576 in mouse, and 996 in humans. The top candidates for each species are given in Table S4.

In order to assess the sensitivity and specificity of this guilt-by-association concept, we applied a leave-one-out test [22]. By omitting one of the DR-essential genes from the seed list and checking whether the removed gene was among the significant candidate genes (i.e. predicted correctly), we obtained a measure for sensitivity of the guilt-by-association method in the form of the percentage of genes which were recovered (i.e. rediscovered). This method achieved a recovery rate of 40% in yeast, 12% in worm and 50% in fruit fly. The lower recovery rate for worms compared to the other species (and the higher p-values) is possibly because *C. elegans* had the lowest number of known interactions at the time of analysis.

**Figure 2:**
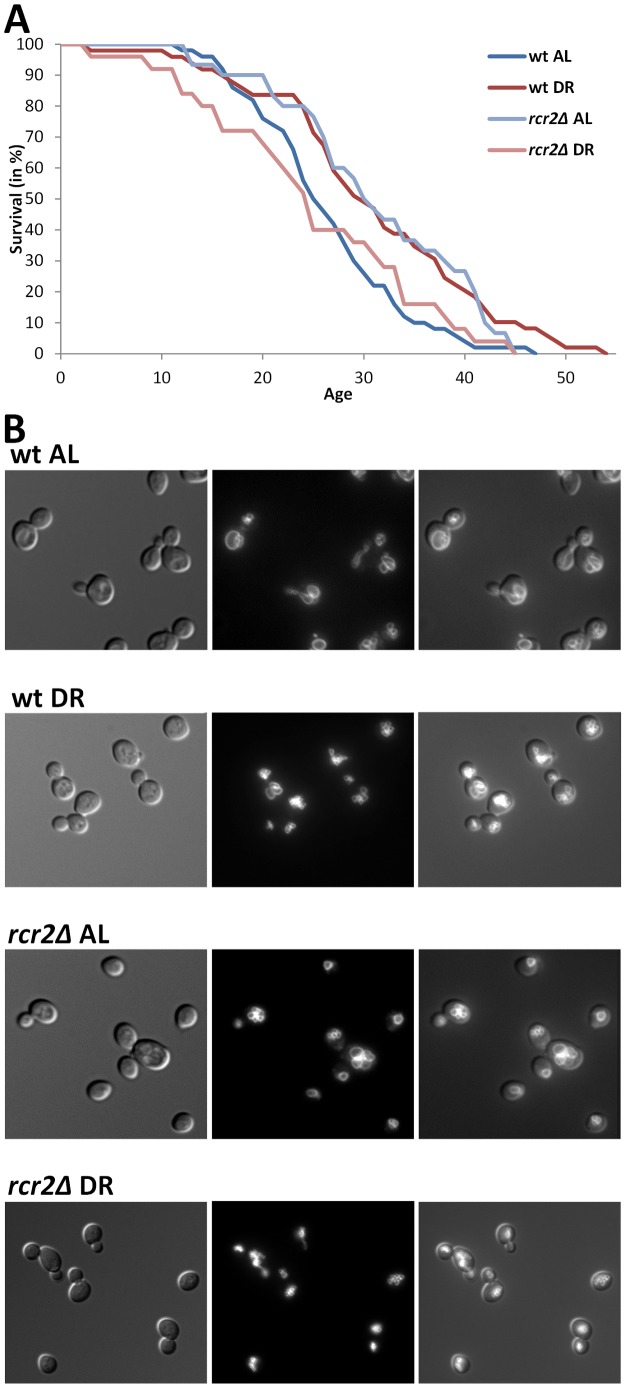
DR–essential vacuole-associated genes are required for DR lifespan extension and normal vacuolar morphology. A, Survival curves of wild-type and *rcr2Δ* mutant strains measured on AL/YEPD and DR media. The units of age (X axis) are generations. Mean and maximum lifespans as well as sample sizes are listed in Table 2. B, Vacuolar morphology on AL and DR media of wild-type and *rcr2Δ*. Left panel: DIC images showing the cells; middle panel: TRITC-fluorescent images showing the vacuoles; right panel: overlay of cell and vacuole images.

To test the new candidate genes, and thus to validate the usefulness of GenDR and DR network analyses as tools for aging research, we experimentally assessed the role in DR of some of our yeast candidates. Vacuole-related terms were among the most significant discrete clusters of terms in the DR-essential network of yeast (Enrichment Score: 5.2; Benjamini p-value, *p*_B_<2·10^−5^). Our guilt-by-association method predicted nine yeast genes whose protein products are on the vacuolar membrane and are non-essential for viability. Therefore, we decided to focus on the mutants of these genes, and measured replicative lifespan and vacuolar morphology of these nine mutants on AL and DR (Table 2 and Figure 2). Eight of the nine mutants we analyzed exhibited an impaired lifespan extension by DR (i.e. are DR-essential genes). Five of the nine mutations altered the extent of DR-induced vacuole fragmentation. While the vacuolar morphology of wild-type and *rcr2Δ* are indistinguishable in AL, their morphologies in DR media are different; wild-type had some cells with a few vacuolar vesicles (equal or less than 5/cell) but *rcr2Δ* cells had 6 or more vacuolar vesicles/cell (Figure 2). Similar to mutants (*erg6Δ*, *nyv1Δ*, etc.) with highly fragmented vacuoles under DR [23], *rcr2Δ* had a shortened lifespan on DR (Figure 2). Deletion of *OPT2* or *FRE6* showed results similar to *rcr2Δ* and all had an extended lifespan on AL (Table 2).

**Table 2:** Lifespan and vacuolar changes of novel DR–essential genes.

Mutant	GbA p-value	Mean/max. AL lifespan, (N)	Mean/max. DR lifespan, (N)	AL, mutant vs. WT, log-rank p-value	DR vs. AL, log-rank p-value	Vacuolar phenotype on AL	Vacuolar phenotype on DR	Vacuolar change	Ratio of mRNA levels (DR/AL) in WT
wild-type	-	26.4/47 (51)	31/54 (50)	-	0.002	round	round	-	-
*vps20Δ*	0.02	22.07/38 (30)	18.27/34 (30)	0.09	0.052	-	-	-	1.56
*fre6Δ*	0.022	30.07/46 (30)	28/42 (30)	0.043	0.173	round	fragmented	29→14	0.85
*rcr2Δ*	0.019	31.27/45 (30)	24.96/45 (25)	0.008	0.048	round	fragmented/round	52→18	3.22 *
*ydl180wΔ*	0.043	27.67/44 (30)	28.2/46 (25)	0.262	0.596	fragmented	fragmented/round	-	2.25 *
*opt2Δ*	0.048	32.6/51 (15)	25.43/39 (30)	0.007	0.015	fragmented	fragmented	-	0.05*
*gtr1Δ*	0.037	16.93/23 (15)	18.73/29 (15)	4.5e-09	0.208	round	fragmented	-	2.77 *
*dap2Δ*	0.013	21.27/30 (15)	19.93/32 (15)	0.001	0.969	round	fragmented	60→1	1.22
*slm4Δ*	0.049	23.87/48 (15)	23.4/36 (15)	0.793	0.597	round	fragmented	44→16	3.28 *
*yol092wΔ*	0.043	20.8/34 (15)	27.4/42 (15)	0.087	0.062	fragmented	fragmented	-	0.33*

Vacuolar-implicated genes, which were found via a guilt-by-association (GbA) strategy using DR-essential genes, were tested in replicative-lifespan assays under *ad libitum* (2% glucose) and DR (i.e. 0.5% glucose) diets. Samples with over 30% of cells having 1 round vacuole/cell were classified as round vacuole and samples with less than 30% of cells having 1 round vacuole were classified as fragmented. Lifespan data is presented in the form a/b (c) where a = mean, b = max., and c = N. p-values are for the mean lifespan comparisons. For vacuolar morphology, about 100–200 cells were counted in each sample. Genes for which the transcript-level change was >2-fold are marked with an asterisk. Vacuolar change = changes of the fraction (%) of cells with 1 round vacuole per cell from AL→DR. WT = wild-type.

### Differential Expression of DR–Essential Genes upon DR

For the purpose of gaining insights into which processes on the global scale are affected by DR, we generated microarray data in yeast under DR (see Materials and Methods) and those genes more than two-fold differentially expressed were examined using DAVID by retrieving terms and clusters with FDR<5%. DR up-regulated genes were associated with heat response, mitochondria, peroxisome, transcription, mRNA processing, zinc binding, carbohydrate metabolism, sporulation, vacuole, and mitochondrial ribosomes, while DR down-regulated genes were related to ribosome/translation, nitrogen, sterol and one-carbon-source metabolism as well as DNA-replication (Table S5).

This comparison of mRNA levels between yeast cells cultured in AL and DR media, as ascertained on microarrays, also furnished further support for the involvement of the predicted vacuolar DR-essential genes. Of the 9 predicted vacuolar DR-essential genes, 6 were differentially regulated by more than two-fold either up or down (Table 2). Strikingly, *OPT2* which had the strongest effect on lifespan when deleted (around 30% mean and maximum lifespan extension), was also among the genes most strongly (20-fold) down-regulated by DR ([24] and our DNA-microarray data; see Table 2).

As changes in expression of DR-essential genes via genetic manipulations mimic and/or abolish the lifespan extension conferred by DR, one interpretation is that DR-essential genes normally mediate DR effects by changing their expression or activity level in response to DR. As an approximation to investigate this, we employed large-scale microarray-expression profiles of yeast, worm [25] and fly [26], [27]. Numerous DR-essential genes change in their activity either at the transcriptional or translational level in response to DR (Tables S6, S7, S8, S9, S10). Of note among genes differentially expressed during DR, only two DR-essential homologous groups are shared in common by yeast, worm and fly: homologs of a fatty-acid elongase, and S-adenosylmethionine synthetase activity (Table S11). Experimental data support the importance of these two categories for which GenDR and DR-induced differential expression agree: (1.) worms with RNAi-suppressed fatty-acid elongase activity have increased lifespan, and long-lived *C. elegans* mutants tend to have shorter fatty-acid chains in proportion to their longevities [28]; and (2.) knockdown of the nematode SAM synthetase gene, *sams-1*, extends adult lifespan [29], [30], and also in yeast *SAM1* deletion extends lifespan [31]. However, DR-essential genes in yeast are no more likely to be differentially expressed than expected by chance (30 of 70 DR-essential genes among the DR-differentially expressed genes; hypergeometric p-value = 0.6) at a threshold of two-fold change.

### Transcription Factors Governing the DR Signatures in Yeast

To unravel the relationship between DR-essential genes and differential expression upon DR we utilized a gene regulatory network (transcription factor – target gene interactions [32], [33]; a subcategory of our integrated network) in yeast to identify candidate transcription factors responsible for the differential expression upon DR. Our criteria were factors which, firstly, regulate DR-differentially expressed genes with a high specificity, and secondly, interact with DR-essential genes either at the physical or genetic level. Transcription factors which had very high specificity (i.e. specific/total interactions, in %) for controlling DR-induced genes included not only factors involved in nutrient-sensing (e.g. Msn2/4 (26%), Gis1 (53%), Mig1 (40%)), and stress response (e.g., Hsf1 (34%)), both of which are known to be important for DR-lifespan extension, but also meiotic transcription factors such as Ime1 (41%), Ume1 (75%), Ume6 (38%) and Ndt80 (26%) (Table S12).

Yeast sequences upstream (within 500 bp) of the promoter regions of genes displaying >2-fold differential expression under DR were examined for motif enrichment (by hypergeometric test) and indeed, upregulated genes are significantly enriched for a number of upstream regulatory elements: STRE (stress-response element), PDS (post-diauxic shift) element, URS1 (upstream regulatory sequence 1), and MSE (middle-sporulation element) including the motifs of Ume6, Ime1 and Ndt80 (Tables S13, S14, S15). Interestingly, URS1 as well as motifs of Ume6, Sum1, Ndt80, and Ime1 are even enriched in the upstream sequences of the full set of DR-essential genes (Table S16). Also, in another signature of DR [24], meiotic transcription factor binding motifs were significantly enriched in the 500 bp upstream regions of DR-induced genes. For instance, YGNCACAAAW (NDT80) was present upstream of 14 genes, of which 11 are DR-induced genes (p-value = 0.0025, q-value = 0.029). NDT80 transcript levels increase as a function of the strength of DR [34] and at 0.5% glucose concentration in our own DNA-microarray data, were found to be 2.6-fold elevated.

### DR–Essential Genes Are Triggered by Lifespan-Extending Spermidine

Comparing different longevity interventions for their commonly regulated processes may allow us to pinpoint the gene expression changes most essential for lifespan extension. Similar to DR, treatment with the polyamine spermidine extends lifespan in multiple model organisms such as yeast, worm, fruit fly, as well as in human cells *in vitro* [35].

We found that, in yeast [36], spermidine treatment causes differential expression of DR-essential genes more often than expected by chance: at a threshold of two-fold change, 15 of the 70 DR-essential genes were among the 727 spermidine-differentially expressed genes (hypergeometric p-value = 0.003). This effect appears to be very specific as there was no enrichment observed among genes induced by spermine, another polyamine, which has not to our knowledge been shown to affect lifespan. Moreover, DR and spermidine signatures regulate an overlapping set of genes (far more likely than expected by chance): at two-fold change, 323 of 727 spermidine-differentially expressed genes were among the 2560 DR-differentially expressed genes (hypergeometric p-value = 4·10^−4^), whereas genes regulated by DR and spermine did not overlap more than expected by chance at any cut-off (e.g. hypergeometric p-value = 0.69 for two-fold changes).

Comparison of the transcriptional signatures of DR (derived from our own DNA-microarray data) and spermidine treatment by looking for common functional enrichment terms among genes differentially expressed with DR and spermidine, indicates that both DR- and spermidine-upregulated genes are highly enriched in *sexual sporulation* (*p*_B_<10^−3^), *peroxisome* (*p*_B_ = 10^−3^), *mitochondrion* (*p*_B_<0.02) and *ubiquitin conjugation pathway* (*p*_B_<10^−3^) and downregulated genes are enriched in *sterol metabolic process* (*p*_B_<0.05) as well as *pentose transmembrane transporter activity* (*p*_B_ = 0.014). The set of common functional terms enriched by both spermidine and DR treatments (at a 1.5-fold-change threshold) revealed that both induce *autophagy*, *heat response* and *sporulation* (Tables 3 and 4). Spermidine treatment leads to both a significant induction and suppression of sporulation/meiosis genes (Table S17) and strikingly similar to DR, induces *NDT80* expression.

**Table 3:** Terms associated with DR and spermidine upregulated genes.

Term	analytic p-value	z-method p-value
GO:0034605∼cellular response to heat	9.18e-13	6.8e-13
GO:0009266∼response to temperature stimulus	8.6e-11	2.23e-10
GO:0009408∼response to heat	4.45e-10	3.48e-10
GO:0006914∼autophagy	7.14e-11	4.12e-09
GO:0009628∼response to abiotic stimulus	2.48e-08	9.56e-08
peroxisome	1.4e-06	2.75e-06
GO:0055114∼oxidation reduction	1.26e-3	1.08e-3
GO:0033554∼cellular response to stress	5.38e-3	3.05e-3
GO:0030437∼ascospore formation	8.39e-3	4.33e-3
GO:0034293∼sexual sporulation	8.39e-3	4.33e-3
GO:0043935∼sexual sporulation resulting in formation of a cellular spore	8.39e-3	4.33e-3
GO:0016042∼lipid catabolic process	7.83e-3	4.94e-3
sporulation	0.001	8.41e-3
GO:0005628∼prospore membrane	0.002	8.74e-3
GO:0042763∼intracellular immature spore	0.002	8.74e-3
GO:0042764∼ascospore-type prospore	0.002	8.74e-3
GO:0043167∼ion binding	0.003	0.002
GO:0043169∼cation binding	0.004	0.002

Specific terms are significantly upregulated in both DR and spermidine treatment.

**Table 4:** Terms associated with DR and spermidine downregulated genes.

Term	analytic p-value	z-method p-value
gpi-anchor	4.05e-08	3.26e-08
GO:0009277∼fungal-type cell wall	2.44e-07	1.43e-07
glycoprotein	3.65e-07	1.88e-07
GO:0005576∼extracellular region	1.63e-06	7.15e-07
glycosylation site:N-linked (GlcNAc…)	1.48e-06	7.55e-07
GO:0031225∼anchored to membrane	2.95e-06	1.5e-06
GO:0005618∼cell wall	3.47e-06	1.81e-06
GO:0030312∼external encapsulating structure	3.47e-06	1.81e-06
cell wall	3.46e-06	1.96e-06
propeptide:Removed in mature form	3.94e-06	2.21e-06
Secreted	1.37e-05	6.9e-06
topological domain:Extracellular	2.36e-05	2.07e-05
cross-link:Glycyl lysine isopeptide (Lys-Gly) (interchain with G-Cter in ubiquitin)	6.44e-3	3.11e-3
isopeptide bond	0.001	6.06e-3
sce00260:Glycine, serine and threonine metabolism	0.001	8.49e-3
cell membrane	0.002	0.001
GO:0006414∼translational elongation	0.002	0.001
ubl conjugation	0.007	0.004

Specific terms are significantly downregulated in both DR and spermidine treatment.

### Comparing DR Interactomes

**Figure 3:**
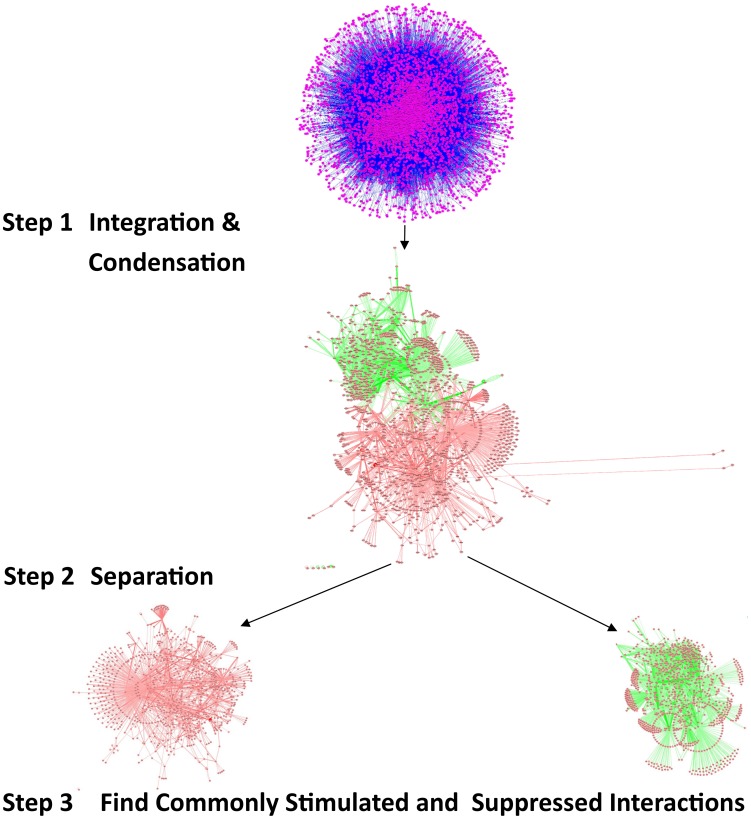
Comparing the influence of gene-expression changes upon DR, in multiple interactomes. The interactomes of yeast, worm, and fly were integrated with gene expression information upon DR and condensed interaction networks generated via the *ExprEssence* algorithm (Step 1). Interaction networks restricted to either suppressed or stimulated interactions for each species were created (Step 2). The suppressed and stimulated networks were then separately compared for common significant functional enrichments (Step 3). Functional terms which were common to suppressed and induced interactions upon DR in yeast, worm, and fly are listed with their respective p-values (Tables 5 and 6).

The validation of vacuole mutants and the responses of DR-essential genes to longevity manipulations support the biological relevance of predictions based on the DR-interactome. To further study conserved mechanisms of DR-induced life-extension, we next integrated and analyzed interactome and transcriptome data (Figure 3). The interactomes of yeast, worm and fly were integrated with their respective DR-triggered gene expression changes and condensed interaction networks were generated via the *ExprEssence* algorithm [37]. These networks were further condensed until the representative graph contained approximately 1,000 genes. Two graphs for each species were created, one containing only startups (induced interactions), while the other contained only shutdowns (suppressed interactions), thus partitioning the interactions of up- and down-regulated genes, respectively. Next, DAVID was used to retrieve the functionally enriched terms for each graph, retaining only the terms common to either all startups or all shutdown networks which surpass a *p*_B_<0.05. Subsequently, p-values were combined across all three species (Tables 5 and 6; see Materials and Methods). Suppressed interactions were enriched for terms associated with translation such as *ribonucleoprotein*, *structural molecule activity* and *ribosome*. This is consistent with previous observations that down-regulation of translation or ribosomes extends lifespan in diverse organisms from yeast to mammals [38]–[41]. Stimulated interactions mostly occurred between genes involved in modifying chromatin structure such as *chromatin regulator*, *chromatin modification*, and *chromosome organization*, genes associated with *reproductive developmental/cellular processes* as well as *cell cycle* and *stress response*. Strikingly, the majority of these cell-cycle-associated genes are involved in *meiosis* with p-values of <10^−19^, <10^−46^, and <10^−5^ for yeast, worm, and fly, respectively (Table S18).

**Table 5:** Terms associated with DR-stimulated interactions in the comparative interactomic changes upon DR.

Term	analytic p-value	z-method p-value
phosphoprotein	1.59e-57	7.36e-43
nucleus	7.21e-52	7.53e-29
serine/threonine-protein kinase	2.04e-37	4.2e-28
GO:0003006∼reproductive developmental process	2.26e-28	1.05e-24
kinase	1.37e-39	1.16e-24
GO:0007049∼cell cycle	9.09e-43	1.46e-23
nucleotide-binding	5.22e-47	3.56e-22
GO:0022402∼cell cycle process	8.01e-36	3.13e-21
IPR002290:Serine/threonine protein kinase	1.21e-26	6.28e-21
GO:0019953∼sexual reproduction	3.29e-28	1.47e-20
atp-binding	4.65e-44	1.02e-19
nucleotide phosphate-binding region:ATP	2.3e-21	2.62e-18
GO:0051276∼chromosome organization	3.19e-18	5.31e-17
chromatin regulator	5.46e-18	6.66e-17
GO:0016568∼chromatin modification	1.67e-18	6.1e-16
GO:0048610∼reproductive cellular process	6.72e-16	1.44e-14
ATP	4.81e-15	2.43e-14
GO:0042802∼identical protein binding	5.67e-13	6.46e-12
GO:0044257∼cellular protein catabolic process	8.27e-12	5.53e-11
coiled coil	1.26e-11	8.29e-11
GO:0010605∼negative regulation of macromolecule metabolic process	7.1e-11	3.43e-10
GO:0010629∼negative regulation of gene expression	6.49e-11	4.07e-10
GO:0005694∼chromosome	1.97e-09	4.98e-09

Defined terms are associated with induced interactions by DR commonly across different species (yeast, worm, and fly).

**Table 6:** Terms associated to DR-suppressed interactions in the comparative interactomic changes upon DR.

Term	analytic p-value	z-method p-value
phosphoprotein	5.96e-103	1.51e-42
cytoplasm	7.74e-79	2.41e-30
nucleus	1.6e-50	3.11e-30
nucleotide-binding	5.01e-47	3.5e-30
ribonucleoprotein	2.62e-92	2.03e-29
GO:0030529∼ribonucleoprotein complex	7.06e-93	7.71e-29
GO:0043228∼non-membrane-bounded organelle	9.01e-88	7.94e-28
GO:0043232∼intracellular non-membrane-bounded organelle	9.01e-88	7.94e-28
GO:0005198∼structural molecule activity	1.15e-34	1.08e-27
atp-binding	4.67e-31	2.29e-25
rna-binding	5.37e-36	8.38e-22
GO:0044445∼cytosolic part	4.58e-56	2.09e-20
ATP	2.28e-20	2.99e-19
ribosome	3.42e-41	4.6e-19
GO:0042802∼identical protein binding	5.09e-16	1.95e-14
GO:0000166∼nucleotide binding	2.06e-14	2.1e-13
ubl conjugation	1.18e-11	5.58e-11

Defined terms are associated with suppressed interactions by DR commonly across different species (yeast, worm, and fly).

In accordance with the functional enrichment analysis of DR-essential networks (Table S3), the comparative interactomics changes suggest that DR both stimulates and suppresses interactions associated with *phosphoproteins* and *nucleus* (Tables 5 and 6). To unravel the relationship between these two broad terms we identified all kinases and phosphoproteins in yeast known to be localized in the nucleus (using Gene Ontology annotation), that are either up- or down-regulated upon DR (Table S19). The induced and suppressed nuclear kinases (Table S20) are significantly enriched for *meiosis* (each *p*_B_<10^−4^; Table S21). While most of the induced nuclear kinases are positive regulators, the suppressed nuclear kinases are mainly negative regulators of meiosis, indicating that DR in yeast promotes the initiation of meiosis. Interestingly, yeast genes associated to *meiosis*, according to Gene Ontology (161 genes), had an overall average gene expression increase of 2.7 upon DR (higher than expected by chance; Mann Whitney U test p-value = 0.002) and together with all their interaction partners were on average 2-fold upregulated (Mann Whitney U-test p = 0.003). Induced nuclear phosphoproteins (i.e. substrates of kinases) are enriched for *protein phosphatases* (*p*_B_<10^−23^), *ER-nuclear signaling* (*p*_B_<10^−4^), *MAPK signaling*, *Mn* (*p*_B_<10^−4^), *sexual reproduction*/*reproductive cellular process* (*p*_B_<6·10^−3^/0.03) (Table S21), while suppressed nuclear phosphoproteins were only enriched for *protein phosphatases* (*p*_B_<10^−10^) (Table S22).

**Figure 4:**
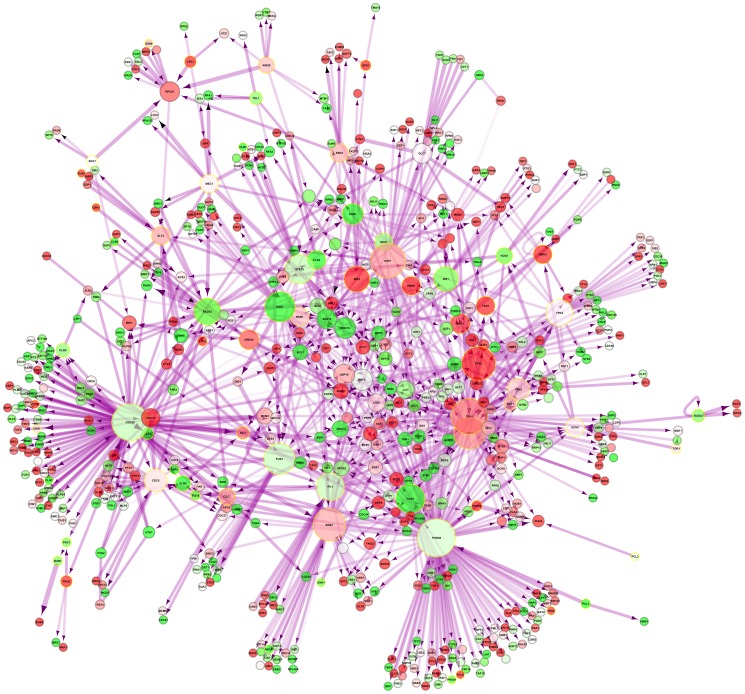
Nuclear phosphorylation network of yeast. DR–upregulated genes are shown in red and downregulated genes in green. Kinases are marked by a golden halo.

An interaction network of these kinases in yeast and their direct interaction partners restricted only to direct physical interactions in the nucleus was generated (Figure 4). *IME2*, which is the second most-upregulated nuclear kinase upon DR (Table S19), acts on the induced transcription factors *IME1* and *NDT80* as well as on the suppressed *SUM1*. In *C. elegans*, induced kinases were also enriched among other terms for *female meiosis* (*p*_B_<0.03; Table S23). In line with the enrichment of reproduction-related terms in the DR-essential network (Table S3) in *Drosophila*, induced kinases restricted to the nucleus were enriched for *female gamete generation*/*sexual reproduction*/*regulation of cell cycle*/*multicellular organism reproduction* (*p*_B_<5·10^−3^/0.02/0.02/0.02; Table S24). Finding upregulation of reproduction-related genes is surprising given the known suppressive effect of DR on reproduction [42].

## Discussion

### A Database and Analysis of Genes Essential for DR Life-Extending Effects

Understanding the genetic basis of DR is of great importance not only to help elucidate mechanisms of aging but also to understand how diet can influence aging, longevity, health, and age-related diseases, which may have major human therapeutic applications [1], [6]. DR-essential genes are no doubt crucial pieces of the puzzle to solve the mechanism of DR [1], [2], [17], but to date they have not been studied as a unified system. To address the need for a more systematic study of the mechanisms of DR-conferred lifespan extension, we created GenDR, the first database of DR-essential genes and indeed the first database of DR-related genes. GenDR provides manually-curated information on genetic mutations which interfere with the pro-longevity effect of DR as well as their respective homologous genes in the major model organisms and humans. Because different genes appear to be important in different regimens and there are conflicting findings regarding some of them, different DR regimens across multiple model organisms are covered, allowing researchers to focus on their preferred system. With the growing importance of network biology, functional genomics and systems biology to study aging and DR [6], GenDR will be a valuable tool for researchers to study the genetic and molecular mechanism of DR as evidenced by our validation of vacuole mutants (Figure 2) and gene regulatory inference (Table S16).

Our study of the molecular evolution of DR-essential genes (Figure 1) showed conservation at the molecular level reflecting the observation that DR extends lifespan in various evolutionarily distant organisms. Interestingly, DR-essential genes had lower dN/dS ratios than expected purely by chance, as well as lower dN and dS values. This could indicate that natural selection has constrained not only the amino acid sequences, but also the nucleotide sequences which may include regulatory elements. The molecular interaction data support the observation that DR-essential genes are conserved across vast evolutionary distances, as genes with relatively high degree, i.e. hubs, are more ancient and evolve more slowly than genes that encode non-hub proteins [43]–[45]. One caveat of this analysis is that these results may also reflect a bias in the selection of genes, as researchers tend to study genes which have orthologs and have been shown to affect DR in other organisms. Similarly, the result that DR-essential genes interact with each other more than expected by chance might also be affected by selection bias, as genes studied in the context of DR are often part of similar or closely interacting biological pathways (e.g. insulin/IGF1 signaling and TOR). Also, both DR-mechanism and aging-effect genes tend to be enriched for signal-transduction genes, especially those which are well known to be highly conserved [46].

### Network Analyses Reveal Novel DR–Essential Genes and Candidate Mechanisms

**Figure 5:**
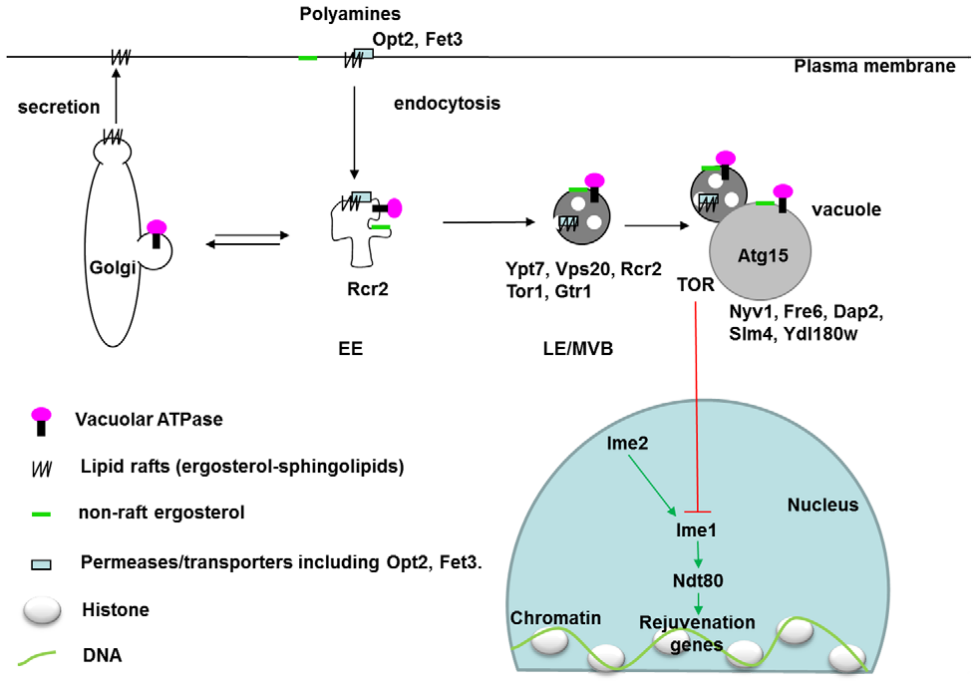
Localization of vacuole-related DR–essential proteins and lipids in yeast cells. In response to environmental triggers such as DR, membrane transporters (Opt2 and others) on the plasma membrane are endocytosed through lipid rafts (ergosterol-sphingolipids) after ubiquitinylation. On early endosomes, proteins and lipids are sorted to late endosomes, Golgi, or other organelles. In late endosomes, different endosomal sorting complexes required for transport (ESCRT) push the target proteins with their membranes into the lumen of late-endosome, or multivesicular bodies (MVB). Matured MVB can fuse with vacuoles, within which proteases and lipases (Atg15) degrade target proteins and lipids. In mammalian cells, maturation of late endosomes is essential for activation of the master regulator for growth, the mammalian target of rapamycin 1 (mTORC1). In yeast cells, TORC1 suppresses meiotic transcription factors which are normally only active during gametogenesis [110]. The inhibitory effects of DR on TORC1 thus activate these rejuvenation genes. EE: early endosome. LE: late endosome. MVB: multivesicular body. Black arrows indicate movement of vesicles. DR-essential proteins are labelled in their localized organelles. Localization data were extracted from the SGD database. Ypt7 localization on late endosomes was reported by [111]. Green arrows indicate activation and a red line indicates an inhibitory effect.

Our network analyses successfully predicted novel DR-essential genes in yeast. This illustrates that it is possible to associate *in silico* genes that are of interest in a DR context, with other, little-known genes, and derive testable new hypotheses. Network analyses based on GenDR have therefore the capacity to implicate new genes related to lifespan extension by DR. In particular, we identified eight novel vacuole-related DR-essential yeast genes that impair DR-mediated longevity when deleted, of which three (*OPT2*, *FRE6,* and *RCR2*) extended lifespan in AL and prevented any further lifespan-extension by DR. Although the genome-wide frequency of DR-essential genes is unknown, finding 8/9 of such genes with 3/9 extending lifespan in AL is likely much more than expected by chance and illustrates the utility of our method. These genes had been previously implicated in drug detoxification (*OPT2*), transition metal ion homeostasis (*FRE6*) and endosomal-vacuolar trafficking of plasma membrane proteins (*RCR2*, *VPS20*, *GTR1*, *SLM4*) as well as a vacuolar membrane protein *DAP2*, plus a protein of unknown function (*YDL180W*). Comparing localizations of the proteins encoded by these genes with the known DR-essential genes in our database revealed endocytosis as one target of DR (Figure 5). Moreover, the different effects on lifespan of mutants missing these newly identified DR-essential genes (Table 2) prompted us to speculate on the function of each endocytic step in longevity.

Cells take up nutrients through plasma-membrane transporters including oligopeptidase transporter Opt2 and ferrous transporter Fet3. Secretion and endocytosis determine the fate of these transporters depending on environmental cues (Figure 5). Both secretion and endocytosis depend on the lipid composition of membranes. Blocking ergosterol synthesis (erg6Δ) halts the degradation of Tat2, the high-affinity transporter of tryptophan, and traps it in late endosomes (or multivesicular bodies) [47]. Nutrient transporters destined for degradation in vacuoles are ubiquitinated by Rsp5 (a DR–essential ortholog of *C. elegans* WWP-1 [48]) on the plasma membrane or endosomes [49]. Ubiquitinated cargoes are sorted to late endosomes, where endosomal sorting complexes required for transport (ESCRT) work sequentially to encapsulate the ubiquitinated transporters within the endosomal lumen. Vps20 is a component of ESCRT III, which seals the membranes [50]. Our identification of *VPS20* as a DR-essential gene may suggest the involvement of late endosomes in longevity. In support of this possibility, a screen for mutants that shorten yeast chronological lifespan also revealed a critical role of the late endosome [51], [52].

For several of the mutants tested the lifespan was shorter than wild-type, which indicates that these genes are required for normal longevity and especially for the response to DR (perhaps due to their involvement in endocytosis). There are also four mutants (*opt2Δ*, *rcr2Δ*, *vps20Δ*, *fre6Δ*) with a shorter lifespan on DR than on AL (Table 2), comparable to the “inverse DR” effect reported for *erg6Δ*, *ypt7Δ*, and *nyv1Δ* mutants [23]. These mutants are all likely to be blocked in the maturation of late endosomes. Deletion of Rcr2 or blockage of ergosterol synthesis (*erg6Δ*) led to accumulation of endocytic vesicles (or a special subset of apparent vacuoles as visualized by the dye FM4-64) that are unable to fuse with other vacuoles, and to the apparent formation of highly fragmented vacuoles (Figure 2B; also see Figure 4B of [23]). The fact that deletion of the plasma membrane transporter *OPT2* also results in fragmented vacuoles [53] may indicate improper fusion between late endosome and vacuoles. The maturation of late endosomes also depends on the proton gradient across the membrane, which is maintained by a membrane H^+^-ATPase. *FRE6* deletion could impair metal-ion efflux and consequently decrease cytosolic iron and copper levels [54]. Copper ions control the activity of membrane H^+^-ATPase activity [55]. As such, endocytosis appears to be essential for normal lifespan under AL and especially for the lifespan-extension response under DR conditions. Endocytosis may for instance remove damaged plasma membrane proteins, and in higher organisms even intercellular debris.

In addition to implicating the plasma-membrane-endosome-vacuole pathway as one target of DR, the longevity of three mutants (*opt2Δ*, *fre6Δ*, an*d rcr2Δ*) under AL (Table 2) reveals a putative novel controlling mechanism of the target of rapamycin complex 1 (TORC1). *OPT2* and *FRE6* exhibit negative genetic interactions with *TOR1* [56] and *TCO89* [57], respectively, both of which encode components of TORC1. A negative genetic interaction usually suggests a cooperative relationship [57]. Rcr2 interacts with Ypt7 in a yeast 2-hybrid assay [58]. While it is not known to be true in yeast, the mammalian ortholog of Ypt7, Rab7, is essential for mammalian TORC1 activation [59]. It is thus tantalizing to speculate that TORC1 may be partially inactivated in *opt2Δ*, *fre6Δ*, or *rcr2Δ* strains. Considering that part of TORC1 is on the vacuolar and endosomal membranes [60], it is not surprising to observe that some vacuolar membrane mutants affect the activity of TORC1. Another beneficial effect of *opt2Δ* in longevity might be the uptake of life-extending polyamines (see also [61]).

### Transcriptional Changes upon DR and Their Regulators

There was only a slight enrichment of DR-essential genes among those differentially expressed during DR. This is not unexpected, since DR-essential genes are likely to lie upstream in the DR-longevity pathway, whereas most genes expressed differentially upon DR would tend to lie downstream in an effector cascade. It is also possible that many DR-essential genes mediate their effects on lifespan via transient expression, changes in protein levels, or in biochemical activities that are not reflected in analyses of mRNA levels. The fact that we did not differentiate between the various DR regimens would also dilute the enrichment of essential genes shared by multiple pathways. Nonetheless, numerous DR-essential genes show strong changes at the transcriptional and/or translational level, which may correspond to a respective change in the activity level, or might represent a compensatory response to changes in regulation at other levels. For instance, the only validated DR-essential gene in mammals *Ghr*, extends lifespan if deleted and is also found to be downregulated upon DR (Table S10) [62]. Among the DR-essential homologs commonly differentially expressed in all examined species were fatty acid elongases, which extend lifespan when downregulated in *C. elegans* [28]. In addition, DR regulates S-adenosylmethionine synthetase activity at the transcriptional/translational level in multiple species; its product, S-adenosylmethionine (SAM), is the methyl donor for most methylation reactions. A common significant interactor of DR-essential genes was S-adenosylhomocysteinase catalyzing the reversible hydrolysis of S-adenosylhomocysteine (SAH), which in turn is a competitive inhibitor of methyltransferases. SAM and SAH levels, acting via DR-essential *NNT1*/*NNMT* encoding nicotinamide-N-methyltransferase [63], would impact NAD^+^ metabolism and consequently NAD^+^-dependent protein deacetylases (sirtuins) which were also common interactors of DR-essential genes. SAM and SAH levels might also exert pervasive effects via the control of DR-essential ergosterol biosynthesis in yeast, sterol modifications in *C. elegans* (influencing dauer formation), DNA methylation in mammals, as well as histone methylation and polyamine synthesis in all species.

It is clear that diet alters epigenetic marks, resulting in an altered chromatin state that affects gene expression [64], [65]. Our study provides evidence pointing to a fundamental role of chromatin in DR. Chromatin organization was among the most enriched categories of DR-essential orthologous networks in mammals, and most strikingly several terms related to chromatin were enriched among DR-stimulated interactions in the interactomes in multiple species (Table 5). Modified chromatin structure has been proposed as an underlying mediator of aging from yeast to humans [66], and is known to be influenced by diet [67]. Taken together, our evidence implies that an altered chromatin state in DR is a major component of its lifespan-extending mechanism, conserved across diverse species.

Clearly DR works via a conserved mechanism, but how does it interfere with the aging process? A phenomenon that is at least equally ancient and conserved is the intergenerational/meiotic reversal of all age-related changes that had accumulated, and the restoration of lifespan for progeny [68]. Yeast rejuvenation occurs during sporulation (the equivalent of the mammalian gametogenesis) [68], when transient activation of Ime1 or Ndt80 rejuvenates cells and resets their age to “zero” [68]. We found that both Ime1 (1.4-fold) and Ndt80 (2.7-fold) are induced upon DR, leading us to speculate that DR may act on aging via a mild, continuous form of “rejuvenation”. This would explain why switching the feeding regimen leads to a change in the state, rather than the rate, of aging – i.e. it lowers the mortality level almost immediately [69].

It was proposed that polyamine biosynthesis may be essential for meiosis and sporulation [70]. Spermidine and/or spermine are, in fact, required for sporulation [71] and transition into meiosis [72]. Polyamine concentration declines with age in yeast and human cells. Supplementation with spermidine increased lifespan in yeast, worms, flies, and human cells, and a polyamine-rich diet (as well as probiotics that induce polyamine production) decreased mortality in mice [73], [74]. Spermidine treatment results in hypoacetylation of H3K9, H3K14, and H3K18, probably via inactivation of histone acetyltransferases. Inactivation of the responsible histone acetyltransferases extended replicative lifespan and suppressed lifespan extension by spermidine. While spermidine treatment leads to global histone hypoacetylation, histones on specific promoters such as those of autophagy-related genes were actually hyperacetylated and induced in expression [35]. Increasing acetylation levels of H4K5 and H4K12 via deletion of DR-essential protein deacetylase *RPD3* also increased lifespan in yeast and *Drosophila*. Glucose and nitrogen regulate the switch from histone deacetylation to acetylation of early meiosis genes [75]. It is interesting to note that spermidine could only effectively extend replicative lifespan in yeast when treatment was initiated at old ages [35], indicating that it works via a single round of light rejuvenation. Strikingly, genes differentially expressed by DR and spermidine treatment have certain chromatin marks in common, including histone acetylations, while on the global scale they lead to opposing effects (data not shown). As such, it appears that spermidine might be a downstream component of the DR-mediated lifespan extension and chromatin remodeling induced by DR awakens meiotic rejuvenation programs. Intriguingly, induction of interactions between genes annotated with *reproduction*-related terms was the key shared feature of the comparison of DR-interactomes from yeast, worms and flies.

### Concluding Remarks

Given the multitude of changes induced by DR, the study of DR-essential genes and their networks is an important new tool to distinguish those genes and processes that are the “drivers” of the life-extending effects of DR from those that are mere “passengers”. Our GenDR database will therefore be valuable for numerous researchers and we demonstrate that DR is suited for a network-guided approach. As anticipated, DR-essential genes are conserved at the molecular level and interact with each other more than expected by chance. Crucially, our work demonstrates how gene network analyses of DR allow the identification of novel candidates that can be tested experimentally. Our results also indicate important roles for genes involved in endosomal/vacuolar trafficking in DR-lifespan extension and provide new intriguing candidates for further studies in yeast such as *OPT2*, *FRE6*, and *RCR2*. Genome-wide expression data provide further clues of which genes and processes are regulated by DR, and their systematic integration in interaction networks was initiated in this study. Moreover, gene-regulatory relationships and sequence-based inference both point to an important role of meiotic transcription factors in DR. Finally, by comparing the effects of DR on the interactomes of multiple species, we discovered that DR suppresses translation while stimulating chromatin reorganization and a defined meiosis-related rejuvenation process, across phyla separated by a billion years of evolution. Many questions still remain. What is the nature of the rejuvenation processes utilized by DR to extend lifespan? What is the exact role of polyamines in DR-induced rejuvenation? Are intracellular polyamine levels increased upon DR? And what factors correspond to the mammalian counterpart(s) of *NDT80*? We hope that our efforts in establishing GenDR will help to unravel the secrets of DR-conferred lifespan extension.

## Materials and Methods

### Database Creation

A list of genes, termed DR-essential genes, which if genetically manipulated (knockout by deletion, or transposition, knockdown by RNA interference, or overexpression of transgenes) interfere with the ability of DR to extend lifespan in model organisms (budding and fission yeast, nematode, fruit fly, and mouse) was compiled from the literature. The focus on genes from genetic manipulations experiments means that the selection procedure for selecting genes related to DR will be more objective and unbiased. Genes were included if they interfere with at least one kind of DR regimen which also includes by definition a shift in the response to food concentration at which lifespan is extended (e.g. *chico* gene). A database (GenDR) was implemented in the relational database management system MySQL and a webpage interface was designed (http://genomics.senescence.info/diet/), as for our other aging-related databases [76]. Each entry in the database contains manually curated comments about literature-based evidence and the reason for inferring an association with DR. If there are conflicting reports for a given gene, our policy is to still include the gene in our database but mention all the conflicting reports and then let visitors make their own mind on how to interpret them. This neutral stance policy is similar to the one we already employ for our GenAge database of aging-related genes. Literature citations and links to PubMed are also given. Further, conserved gene expression changes upon DR in mammals are included, although these have been described in another work [62].

### Molecular Evolution

#### Assembling orthologs

Homologs of genes in the GenDR database were retrieved from the NCBI HomoloGene Database, Ensembl/Biomart, OrthoMCL [77], and InParanoid [78] by merging. Genes are either homologous or not, it is a binary assignment, therefore the union was taken by merging them. For the molecular evolution part specifically only Biomart (Ensembl Genes 57) was used to retrieve tables of orthologs (homologs between two different species) for *Saccharomyces cerevisiae*, *Caenorhabditis elegans*, *Drosophila melanogaster*, *Mus musculus*, *Rattus norvegicus*, *Macca mulatta* and *Homo sapiens*. Information about *Saccharomyces pombe* is not available in Ensembl and this species was therefore excluded from the following analyses. For calculating the presence of orthologs, Ensembl protein identifiers were employed to filter for only protein-coding genes. For a given set of genes, here DR-essential genes, it was counted how many of these genes in one species have also at least one protein-coding homologous gene in all the other species under study. This was also done for all protein-coding genes in each genome and given these two values the percentage of presence of homologs were estimated. All pairwise comparisons for each seed species were averaged and the Poisson p-values calculated.

#### Calculating average dN, dS, and dN/dS ratios

Non-synonymous (dN) and synonymous (dS) nucleotide substitution rates [10], [79] were retrieved from the Ensembl Compara built 65 for the orthologous pairs between a rodent (*M. musculus*), a non-human primate (*M. mulatta*) and humans (*H. sapiens*). Orthologs relationships were restricted to the top-hits of InParanoid. In this sense, we corrected for the number of paralogs which might be a source of bias as big gene families tend to have higher dN/dS ratios. The average values for dN and dS as well as dN/dS ratios for the orthologous relationships of DR-essential genes were compared against those without orthologs of DR-essential genes. In order to avoid biases due to overrepresentation of ancient genes, only genes which had also orthologs in lower model organisms (*S. cerevisiae*, *C. elegans*, or *D. melanogaster*) were selected. Thus, the comparison was between ancient genes which are presumably essential for DR versus ancient genes with no known essential role in DR. The corresponding p-values for differences in dN, dS, and dN/dS were calculated with a Mann Whitney U test.

### Molecular Interaction Information

#### Integration

Interactions datasets were retrieved from IntAct (Download 23.01.2011; http://www.ebi.ac.uk/intact/) [80], DIP (Update 10.10.2010) [81], MINT (Version 2010-12-15 updated on 21/12/2010 11:07:00) [82], BIND (http://baderlab.org/BINDTranslation) [83], BioGRID (Release 3.1.72) [84], MPACT [85], DroID (Version 2010_08), Reactome [86], HPRD (Version 9) [87], PDZBase [88], CORUM [89], iRefIndex (05182010) [90], PhosphoSitePlus (Fri Dec 10 13:43:52 EST 2010) [91], PhosphoGRID [92], I2D (Version 1.8) [93], InteroPORC [94], InterologFinder [95], MiMI [96], [97], PINA (Update March 4, 2010) [98], YeastNet [99], WormNet [100], MouseNet [101], and TF-Atlas [102] preferentially as PSI-MAT or simple tab-delimited flat file format. Interactions were aggregated by using a unified schema consisting of synonyms for each gene/protein, their taxonomy identifiers, interaction type and detection system, PubMed identifiers for references, as well as source databases. For simplification, interactions between molecules other than genes, transcripts, or proteins were omitted and only interactions between genes/transcripts/proteins of the same species were considered.

#### Mapping and merging

Symbols, names, aliases and various identifiers of genes, transcripts and proteins were in the first instance mapped to unique Entrez Gene IDs, Ensembl Gene IDs, UniProt IDs and species-specific IDs (from WormBase, MGI, etc.). Ensembl Gene, UniProt, or species-specific database IDs were if possible converted into Entrez Gene IDs. In a second iteration collected synonyms from Ensembl, UniProt, and species-specific database of genes/proteins with missing Entrez Gene IDs were used as queries to map them to synonymous tables of Entrez. In each mapping the Identifier used had always the highest level of matches. For instance, Entrez Gene IDs were chosen with the highest consensus of all other database-derived synonymous tables.

The mapped interactions were merged to a directional merged interactome if the source and target interactors are the same as well as to an undirectional merged interactome in which all interactions describing the same interacting entities are fused regardless of source or target interactors.

For the integrated, mapped and merged interactions a suitable scoring system was developed. Interactions gain scores for each experimental system type, different interaction detection method that was used, and additionally for each different publication which observed an interaction. Furthermore, if an interaction involves a post-translational modification event it receives a further score.

### Network Analyses

The integrated interaction data was transferred into a rational database, queried with gene lists and networks visualized via custom Python scripts or with the use of Cytoscape [103].

Each gene (candidate) was assessed for the number of interactions (degree) with genes in the query lists (seeds), as well as their total interaction number. As a measure of specificity, we calculated the ratio of specific interaction number with the seeds divided by the total number of interactions for each gene (seeds and candidates), as a percentage, and determined its corresponding p-value (based on the binomial distribution). Genes with a binomial p-value<0.05 were classified as significant interactors. Whether a set of genes has a higher average degree than expected by chance was determined using a Mann Whitney U test. We applied an exhaustively leave-one-out test by repeatedly omitting single genes from the seed list and tested whether the genes were among the significant candidate genes inferred from the remainder. From this procedure the percentage of retrieval was estimated for each of the seed genes by successively omitting each gene, one at a time, from the seed list.

### Correlation of Variables and Multiple Linear Regression

We performed a multiple linear regression analysis focused on human genes which considers three variables: the degree of each gene in the interaction network, the number of species with orthologs, and the mean of its dN/dS ratios with three other mammalian species (*M. musculus*, *R. norvegicus* and *M. mulatta*). Genes with a degree of zero or with no given dN/dS ratios were removed, in order to log transform the data. A Pearson correlation was applied to first check whether any of those variables are correlated with each other. Then we made a regression analysis, by the method of least squares, of each variable against other variables first singly and then for a given variable as a function of the other two. The derived linear equations were used to predict the variables of greatest relevance for the set of DR-essential genes.

### Functional Enrichment Analyses

By a FDR of <5% gene lists were examined using the DAVID bioinformatics resource (http://niaid.abcc.ncifcrf.gov/), under default parameters, to identify overrepresented categories [104]. Gene ontologies were retrieved from Entrez and gene descriptions for yeast genes from Saccharomyces Genome Database (SGD; http://yeastgenome.org/) [105].

### Combining p-Values

To judge the overall significance of a series of experiments, the p-values were combined via two different approaches.

The first method is an analytic solution of the Fisher's test [106]. Briefly, the set point whose probability is equal to that of a set of p-values is the hyperbola:


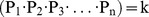


k is the product of a set of p-values k = (x_1_ · x_2_ · x_3_ · … · x_n_), provided that the events described by these p-values are independent of one another. The volume under the surface gives the probability of obtaining a set of p-values as extreme or more extreme than the given set:


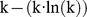


The alternative approach is the z-method as previously described [107], which gives similar, but somewhat more stringent results.

### Lifespan and Vacuolar Morphology Analyses

The replicative lifespan of yeast mutants in the BY4742 (Mat α; his3Δ1; leu2Δ0; lys2Δ0; ura3Δ0) background were measured on different media as described before [23]. The *ad libitum* (AL) medium was YEPD: yeast extract (1%), peptone (2%), agar (2%), and D-glucose (2%). The dietary restricted (DR) medium was yeast extract (1%), peptone (2%), agar (2%), and D-glucose (0.5%). For bud-counting, cells were grown at 30°C and dissected every 100 min. Five to six rounds of bud-counting were performed each day. The aging assay plate was saved at 4°C for overnight and the bud-counting was continued the next day till all cells died, which was diagnosed by either cell lysis or no budding in 2 days. The vacuolar morphology of yeast mutants under AL and DR were measured by FM4-64 labeling and chasing [108]. Briefly, cells were inoculated into AL or DR liquid media (AL: yeast extract (1%) peptone plus (2%) D-glucose; DR: yeast extract (1%) peptone plus (0.5%) D-glucose) and incubated on a shaker (150 rpm) at 30°C overnight. An aliquot of 250 µl of cells were labeled with 6 µl of 2 mg/ml FM4-64 for 1 hour. After washing off the dye, cells were chased in the corresponding medium for 3 hours and then photographed.

Significance of mean lifespan changes were assessed with the log-rank test (a.k.a. Mantel–Cox test) between mutants and wild-type as well as between dietary regimes for each genotype.

### RNA Isolation and Microarray Analyses

Wild-type yeast cells (BY4742) were inoculated into AL or DR liquid media and incubated overnight on a shaker. These seed cultures were then inoculated into fresh media to an initial OD_600_ of 0.01. Cells were grown at 30°C for 12 hours. The final OD_600_ of cultures were 0.85–0.99. Three OD_600_ units of cells were collected and lysed with zymolyase. Total RNA was isolated using the Qiagen RNeasy mini kit as described [109]. Total RNA obtained was between 60 to 100 µg. RNA samples were processed and hybridized with GeneChip Yeast genome 2.0 arrays (Affymetrix Inc., Santa Clara, CA) by Expression Analysis (Durham, NC). The hybridization signals were normalized and analyzed with the Affymetrix statistical algorithms by Expression Analysis. By a two-fold cut-off, out of 5716 probed genes 2587 (45.26%) were differentially expressed, with 1413 genes (24.72%) upregulated and 1174 genes (20.54%) downregulated. Full results and raw microarray data are available online at GenDR (http://genomics.senescence.info/diet/Yeast_array.zip), GEO (GSE38635), and ArrayExpress (E-MTAB-1165).

For additional microarray data from yeast (GSE9217), worms (GSE9682), and flies (GSE26726 and GSE16738), GSE files were retrieved from the GEO database, replicates were averaged and fold-changes calculated via custom Python scripts.

### Transcription Factor Identification

Transcription factor–target gene interactions were retrieved from YEASTRACT (http://yeastract.com/). Transcription factors interacting with DR-essential genes were identified and ordered by their specificity or binominal p-value of the ratio of regulated genes with more than two-fold differentially expression upon DR relative to the total number of genes regulated by the same transcription factor. The whole genome (sacCer3) was retrieved from UCSC and regulatory regions (+500 bp sequences from the transcription start site) annotated. Transcription factor binding motifs were integrated from SGD, YEASTRACT, and YFTD (http://biochemie.web.med.uni-muenchen.de/YTFD/YTF_alpha_2.htm), as well as from the literature. Via regular expression it was tested (hypergeometric p-value and FDR q-values) whether a defined motif is significant enriched in the regulatory regions of differential expressed (either up- or down-regulated) genes or set of genes.

## Supporting Information

Figure S1 DR-essential genes have low dN/dS ratios. Mammalian DR-essential orthologs have lower dN/dS ratio than expected by chance.
(TIF)

Figure S2 **DR-essential gene orthologs in humans form a scale-free network.** Degree distribution of human ortholog-complemented DR-essential gene network, as a log-log plot: i.e. log[degree (k)] is plotted against the log of the number of nodes with degree k (n).
(TIF)

Figure S3 **DR-essential genes are more conserved than aging-related genes.** DR-essential genes have a higher abundance of orthologs than aging-related genes.
(TIF)

Figure S4 **DR-essential genes have a higher node degree than aging-related and signaling genes.** DR-essential genes exhibit a high average node degree relative to aging-related genes or signaling genes.
(TIF)

Table S1 **Mammalian DR-essential orthologs have lower dN/dS ratio than expected by chance.** For each species pair the non-synonymous (dN) and synonymous (dS) substitution rate as well as their ratio (dN/dS) are given for both DR-essential gene orthologs and other conserved genes as control.
(XLS)

Table S2 **Comparing DR-essential genes and aging genes for enrichment in signaling genes.** The enrichment of signaling genes among the DR-essential and aging genes are compared to each other and assessed via a hypergeometric test.
(XLS)

Table S3 **DR-essential gene/orthologs networks are functionally enriched for phosphorylation signaling.** A, For each species the significant genes were analyzed for enrichment of functional terms (OC = Orthologous Complemented). B, Significant functional terms shared by all the species from A.
(XLS)

Table S4 **Top ten DR-essential gene candidates.** DR-essential gene candidates were predicted by using as seeds either DR-essential genes (A) or DR-essential genes complemented with orthologs of DR-essential genes from other species (B).
(XLS)

Table S5 **Enriched clusters for yeast DR-differentially expressed genes.** Clusters of functional terms were retrieved for either all differentially expressed (A), induced (B), or suppressed (C) genes upon DR.
(XLS)

Table S6 **Yeast differential expressed DR-essential genes, orthologs and paralogs.** Yeast DR-essential genes, yeast orthologs of DR-essential genes, and DR-essential gene paralogs which exhibit more than 2-fold change on the transcript level upon moderate DR (0.5% Glucose) are listed.
(XLS)

Table S7 **Worm differentially expressed DR-essential genes and homologs.** Worm DR-essential genes which exhibit more than 1.5-fold change on the transcriptional level upon DR (intermittent fasting) are listed.
(XLS)

Table S8 **Fly differentially expressed DR-essential genes and homologs.** Fruit fly DR-essential genes and homologs which exhibit more than 1.2-fold change on the transcript level at the age of 10 (A) or 40 days (B) are listed.
(XLS)

Table S9 **Fly translational differentially expressed DR-essential genes and homologs.** DR-essential genes and homologs in fruit fly which exhibit more than 1.5-fold change on the translational level are listed.
(XLS)

Table S10 **Mammalian DR-differentially expressed DR-essential genes and orthologs.** DR-essential genes and orthologs in mammals which were commonly differentially expressed across different tissues upon DR are listed. The enrichment of differential expression is indicated as + (upregulated), −(downregulated) and +/− (both and up- and downregulated).
(XLS)

Table S11 **Commonly DR-differentially expressed DR-essential genes across species-boundaries.** Orthologous groups which are commonly differentially expressed upon DR in yeast, worm, and fly are listed.
(XLS)

Table S12 **Yeast transcription factors regulating DR-differentially expressed genes.** Transcription factors with highest specificity to regulate DR-induced (A) or DR-suppressed genes, as well as those which have the highest significance for regulating DR-induced (C) or DR-suppressed (D) genes are listed. Regulatory, physical and genetic p-values correspond to the p-value found in a gene-regulatory, physical-interaction, and genetic-interaction network, respectively.
(XLS)

Table S13 *cis* **-regulatory motif enrichment in yeast DR-differentially expressed genes.** Motifs corresponding to transcription factors were used to scan the genome for the presence within the 500 bp upstream regions of DR-differential expressed genes.
(XLS)

Table S14 ***cis*-regulatory motif enrichment in yeast DR-induced genes.** Motifs corresponding to transcription factors were used to scan the genome for the presence within the 500 bp upstream regions of DR-induced genes.
(XLS)

Table S15 *cis* **-regulatory motif enrichment in yeast DR-suppressed genes.** Motifs corresponding to transcription factors were used to scan the genome for the presence within the 500 bp upstream regions of DR-suppressed genes.
(XLS)

Table S16 **Motifs enriched within 500 bp upstream of the transcriptional start site of DR-essential genes.** Motifs corresponding to transcription factors were used to scan the genome for the presence within the 500 bp upstream regions of DR-essential genes.
(XLS)

Table S17 **Clusters of functional enrichment for spermidine-differentially expressed genes.** Genes which were more than 1.5-fold differential expressed (A: induced; B: suppressed) upon spermidine treatment were used to find for clusters of functional enrichment.
(XLS)

Table S18 **Cell cycle-related genes stimulated by DR in multiple species are enriched for meiosis.** Genes which are belonging to the DR-stimulated interactions (in yeast, worm, and fly) and found to be related to cell-cycle were analyzed for functional enrichment.
(XLS)

Table S19 **DR-induced and suppressed nuclear kinases and phosphoproteins in yeast.** Nuclear kinases as well as phosphoproteins in yeast with their respective ratio of expression changes upon DR are listed.
(XLS)

Table S20 **Yeast nuclear protein kinases expression ratios and descriptions.** Yeast genes annotated to encode protein kinases and to be localized in the nucleus according to Gene Ontology are listed with their respective expression change ratio upon DR and description.
(XLS)

Table S21 **Clusters of functional enrichment for nuclear protein kinases in yeast.** Clusters of functional enriched terms of yeast genes which encode protein kinases and are localized in the nucleus according to Gene Ontology are listed.
(XLS)

Table S22 **Yeast nuclear phosphoprotein expression ratios and descriptions.** Yeast genes annotated to encode phosphoproteins and to be localized in the nucleus according to Gene Ontology are listed with their respective expression change ratio upon DR and description.
(XLS)

Table S23 **Clusters of functional enrichment for worm DR-induced protein kinases.** Clusters of functional enrichment for DR-induced protein kinases in the nematode are listed.
(XLS)

Table S24 **Fly DR-induced nuclear protein kinases functional enrichment.** Clusters of functional enrichment of DR-induced protein kinases in the fruit fly are listed.
(XLS)

## References

[1] Bishop NA, Guarente L (2007) Genetic links between diet and lifespan: shared mechanisms from yeast to humans. Nat Rev Genet 8: 835–844. 17909538 10.1038/nrg2188

[2] Fontana L, Partridge L, Longo VD (2010) Extending healthy life span–from yeast to humans. Science 328: 321–326. 20395504 10.1126/science.1172539 PMC3607354

[3] Kemnitz JW (2011) Calorie restriction and aging in nonhuman primates. ILAR J 52: 66–77. 21411859 10.1093/ilar.52.1.66 PMC3278796

[4] Willcox BJ, Willcox DC, Todoriki H, Fujiyoshi A, Yano K, et al (2007) Caloric restriction, the traditional Okinawan diet, and healthy aging: the diet of the world's longest-lived people and its potential impact on morbidity and life span. Ann N Y Acad Sci 1114: 434–455. 17986602 10.1196/annals.1396.037

[5] Spindler SR (2010) Caloric restriction: from soup to nuts. Ageing Res Rev 9: 324–353. 19853062 10.1016/j.arr.2009.10.003

[6] de Magalhaes JP, Wuttke D, Wood SH, Plank M, Vora C (2012) Genome-environment interactions that modulate aging: powerful targets for drug discovery. Pharmacol Rev 64: 88–101. 22090473 10.1124/pr.110.004499 PMC3250080

[7] Lakowski B, Hekimi S (1998) The genetics of caloric restriction in Caenorhabditis elegans. Proc Natl Acad Sci U S A 95: 13091–13096. 9789046 10.1073/pnas.95.22.13091 PMC23719

[8] Partridge L, Piper MD, Mair W (2005) Dietary restriction in Drosophila. Mech Ageing Dev 126: 938–950. 15935441 10.1016/j.mad.2005.03.023

[9] Barabasi AL, Gulbahce N, Loscalzo J (2011) Network medicine: a network-based approach to human disease. Nat Rev Genet 12: 56–68. 21164525 10.1038/nrg2918 PMC3140052

[10] de Magalhaes JP, Church GM (2007) Analyses of human-chimpanzee orthologous gene pairs to explore evolutionary hypotheses of aging. Mech Ageing Dev 128: 355–364. 17459455 10.1016/j.mad.2007.03.004 PMC2288694

[11] Budovsky A, Abramovich A, Cohen R, Chalifa-Caspi V, Fraifeld V (2007) Longevity network: construction and implications. Mech Ageing Dev 128: 117–124. 17116322 10.1016/j.mad.2006.11.018

[12] Wang J, Zhang S, Wang Y, Chen L, Zhang XS (2009) Disease-aging network reveals significant roles of aging genes in connecting genetic diseases. PLoS Comput Biol 5: e1000521 doi:10.1371/journal.pcbi.1000521. 19779549 10.1371/journal.pcbi.1000521 PMC2739292

[13] Promislow DE (2004) Protein networks, pleiotropy and the evolution of senescence. Proc Biol Sci 271: 1225–1234. 15306346 10.1098/rspb.2004.2732 PMC1691725

[14] Witten TM, Bonchev D (2007) Predicting aging/longevity-related genes in the nematode Caenorhabditis elegans. Chem Biodivers 4: 2639–2655. 18027377 10.1002/cbdv.200790216

[15] Fortney K, Kotlyar M, Jurisica I (2010) Inferring the functions of longevity genes with modular subnetwork biomarkers of Caenorhabditis elegans aging. Genome Biol 11: R13. 20128910 10.1186/gb-2010-11-2-r13 PMC2872873

[16] Bonkowski MS, Rocha JS, Masternak MM, Al Regaiey KA, Bartke A (2006) Targeted disruption of growth hormone receptor interferes with the beneficial actions of calorie restriction. Proc Natl Acad Sci U S A 103: 7901–7905. 16682650 10.1073/pnas.0600161103 PMC1458512

[17] Gems D, Pletcher S, Partridge L (2002) Interpreting interactions between treatments that slow aging. Aging Cell 1: 1–9. 12882347 10.1046/j.1474-9728.2002.00003.x

[18] de Magalhaes JP, Budovsky A, Lehmann G, Costa J, Li Y, et al (2009) The Human Ageing Genomic Resources: online databases and tools for biogerontologists. Aging Cell 8: 65–72. 18986374 10.1111/j.1474-9726.2008.00442.x PMC2635494

[19] Shmookler Reis RJ, Ayyadevara S, Crow WA, Lee T, Delongchamp RR (2011) Gene Categories Differentially Expressed in C. elegans Age-1 Mutants of Extraordinary Longevity: New Insights From Novel Data-Mining Procedures. J Gerontol A Biol Sci Med Sci 67: 366–75. 22021389 10.1093/gerona/glr186

[20] Tazearslan C, Ayyadevara S, Bharill P, Shmookler Reis RJ (2009) Positive feedback between transcriptional and kinase suppression in nematodes with extraordinary longevity and stress resistance. PLoS Genet 5: e1000452 doi:10.1371/journal.pgen.1000452. 19360094 10.1371/journal.pgen.1000452 PMC2661368

[21] de Magalhaes JP, Toussaint O (2004) GenAge: a genomic and proteomic network map of human ageing. FEBS Lett 571: 243–247 doi:10.1371/journal.pgen.1000452. 15280050 10.1016/j.febslet.2004.07.006

[22] Owen AB, Stuart J, Mach K, Villeneuve AM, Kim S (2003) A gene recommender algorithm to identify coexpressed genes in C. elegans. Genome Res 13: 1828–1837. 12902378 10.1101/gr.1125403 PMC403774

[23] Tang F, Watkins JW, Bermudez M, Gray R, Gaban A, et al (2008) A life-span extending form of autophagy employs the vacuole-vacuole fusion machinery. Autophagy 4: 874–886. 18690010 10.4161/auto.6556

[24] Lin SJ, Kaeberlein M, Andalis AA, Sturtz LA, Defossez PA, et al (2002) Calorie restriction extends Saccharomyces cerevisiae lifespan by increasing respiration. Nature 418: 344–348. 12124627 10.1038/nature00829

[25] Honjoh S, Yamamoto T, Uno M, Nishida E (2009) Signalling through RHEB-1 mediates intermittent fasting-induced longevity in C. elegans. Nature 457: 726–730. 19079239 10.1038/nature07583

[26] Bauer J, Antoch M, Chang C, Schorl C, Kolli S, et al (2010) Comparative transcriptional profiling identifies takeout as a gene that regulates life span. Aging (Albany NY) 2: 298–310. 20519778 10.18632/aging.100146 PMC2898020

[27] Zid BM, Rogers AN, Katewa SD, Vargas MA, Kolipinski MC, et al (2009) 4E-BP extends lifespan upon dietary restriction by enhancing mitochondrial activity in Drosophila. Cell 139: 149–160. 19804760 10.1016/j.cell.2009.07.034 PMC2759400

[28] Shmookler Reis RJ, Xu L, Lee H, Chae M, Thaden JJ, et al (2011) Modulation of lipid biosynthesis contributes to stress resistance and longevity of C. elegans mutants. Aging (Albany NY) 3: 125–147. 21386131 10.18632/aging.100275 PMC3082008

[29] Hansen M, Hsu AL, Dillin A, Kenyon C (2005) New genes tied to endocrine, metabolic, and dietary regulation of lifespan from a Caenorhabditis elegans genomic RNAi screen. PLoS Genet 1: e17. doi:10.1371/journal.pgen.0010017. 10.1371/journal.pgen.0010017 PMC1183531 16103914

[30] Steinkraus KA, Smith ED, Davis C, Carr D, Pendergrass WR, et al (2008) Dietary restriction suppresses proteotoxicity and enhances longevity by an hsf-1-dependent mechanism in Caenorhabditis elegans. Aging Cell 7: 394–404. 18331616 10.1111/j.1474-9726.2008.00385.x PMC2709959

[31] Smith ED, Tsuchiya M, Fox LA, Dang N, Hu D, et al (2008) Quantitative evidence for conserved longevity pathways between divergent eukaryotic species. Genome Res 18: 564–570. 18340043 10.1101/gr.074724.107 PMC2279244

[32] Balaji S, Babu MM, Iyer LM, Luscombe NM, Aravind L (2006) Comprehensive analysis of combinatorial regulation using the transcriptional regulatory network of yeast. J Mol Biol 360: 213–227. 16762362 10.1016/j.jmb.2006.04.029

[33] Abdulrehman D, Monteiro PT, Teixeira MC, Mira NP, Lourenco AB, et al (2011) YEASTRACT: providing a programmatic access to curated transcriptional regulatory associations in Saccharomyces cerevisiae through a web services interface. Nucleic Acids Res 39: D136–140. 20972212 10.1093/nar/gkq964 PMC3013800

[34] Lee YL, Lee CK (2008) Transcriptional response according to strength of calorie restriction in Saccharomyces cerevisiae. Mol Cells 26: 299–307. 18679056

[35] Eisenberg T, Knauer H, Schauer A, Buttner S, Ruckenstuhl C, et al (2009) Induction of autophagy by spermidine promotes longevity. Nat Cell Biol 11: 1305–1314. 19801973 10.1038/ncb1975

[36] Chattopadhyay MK, Chen W, Poy G, Cam M, Stiles D, et al (2009) Microarray studies on the genes responsive to the addition of spermidine or spermine to a Saccharomyces cerevisiae spermidine synthase mutant. Yeast 26: 531–544. 19688718 10.1002/yea.1703 PMC3490486

[37] Warsow G, Greber B, Falk SS, Harder C, Siatkowski M, et al (2010) ExprEssence - Revealing the essence of differential experimental data in the context of an interaction/regulation network. BMC Syst Biol 4: 164. 21118483 10.1186/1752-0509-4-164 PMC3012047

[38] Steffen KK, MacKay VL, Kerr EO, Tsuchiya M, Hu D, et al (2008) Yeast life span extension by depletion of 60s ribosomal subunits is mediated by Gcn4. Cell 133: 292–302. 18423200 10.1016/j.cell.2008.02.037 PMC2749658

[39] Hansen M, Taubert S, Crawford D, Libina N, Lee SJ, et al (2007) Lifespan extension by conditions that inhibit translation in Caenorhabditis elegans. Aging Cell 6: 95–110. 17266679 10.1111/j.1474-9726.2006.00267.x

[40] Kapahi P, Zid BM, Harper T, Koslover D, Sapin V, et al (2004) Regulation of lifespan in Drosophila by modulation of genes in the TOR signaling pathway. Curr Biol 14: 885–890. 15186745 10.1016/j.cub.2004.03.059 PMC2754830

[41] Selman C, Tullet JM, Wieser D, Irvine E, Lingard SJ, et al (2009) Ribosomal protein S6 kinase 1 signaling regulates mammalian life span. Science 326: 140–144. 19797661 10.1126/science.1177221 PMC4954603

[42] Shanley DP, Kirkwood TB (2000) Calorie restriction and aging: a life-history analysis. Evolution 54: 740–750. 10937249 10.1111/j.0014-3820.2000.tb00076.x

[43] Fraser HB, Hirsh AE, Steinmetz LM, Scharfe C, Feldman MW (2002) Evolutionary rate in the protein interaction network. Science 296: 750–752. 11976460 10.1126/science.1068696

[44] Eisenberg E, Levanon EY (2003) Preferential attachment in the protein network evolution. Phys Rev Lett 91: 138701. 14525344 10.1103/PhysRevLett.91.138701

[45] Saeed R, Deane CM (2006) Protein protein interactions, evolutionary rate, abundance and age. BMC Bioinformatics 7: 128. 16533385 10.1186/1471-2105-7-128 PMC1431566

[46] Kim SK (2007) Common aging pathways in worms, flies, mice and humans. J Exp Biol 210: 1607–1612. 17449826 10.1242/jeb.004887

[47] Umebayashi K, Nakano A (2003) Ergosterol is required for targeting of tryptophan permease to the yeast plasma membrane. J Cell Biol 161: 1117–1131. 12810702 10.1083/jcb.200303088 PMC2172991

[48] Carrano AC, Liu Z, Dillin A, Hunter T (2009) A conserved ubiquitination pathway determines longevity in response to diet restriction. Nature 460: 396–399. 19553937 10.1038/nature08130 PMC2746748

[49] Nikko E, Pelham HR (2009) Arrestin-mediated endocytosis of yeast plasma membrane transporters. Traffic 10: 1856–1867. 19912579 10.1111/j.1600-0854.2009.00990.x PMC2810449

[50] Babst M (2011) MVB vesicle formation: ESCRT-dependent, ESCRT-independent and everything in between. Curr Opin Cell Biol 23: 452–457. 21570275 10.1016/j.ceb.2011.04.008 PMC3148405

[51] Fabrizio P, Hoon S, Shamalnasab M, Galbani A, Wei M, et al (2010) Genome-Wide Screen in Saccharomyces cerevisiae Identifies Vacuolar Protein Sorting, Autophagy, Biosynthetic, and tRNA Methylation Genes Involved in Life Span Regulation. PLoS Genet 6: e1001024 doi:10.1371/journal.pgen.1001024. 20657825 10.1371/journal.pgen.1001024 PMC2904796

[52] Longo VD, Nislow C, Fabrizio P (2010) Endosomal protein sorting and autophagy genes contribute to the regulation of yeast life span. Autophagy 6: 1227–1228. 20953148 10.4161/auto.6.8.13850

[53] Aouida M, Khodami-Pour A, Ramotar D (2009) Novel role for the Saccharomyces cerevisiae oligopeptide transporter Opt2 in drug detoxification. Biochem Cell Biol 87: 653–661. 19767828 10.1139/o09-045

[54] Sharma PK, Mittal N, Deswal S, Roy N (2011) Calorie restriction up-regulates iron and copper transport genes in Saccharomyces cerevisiae. Mol Biosyst 7: 394–402. 21031176 10.1039/c0mb00084a

[55] Kakinuma Y, Ohsumi Y, Anraku Y (1981) Properties of H+-translocating adenosine triphosphatase in vacuolar membranes of SAccharomyces cerevisiae. J Biol Chem 256: 10859–10863. 6116710

[56] Chan TF, Carvalho J, Riles L, Zheng XF (2000) A chemical genomics approach toward understanding the global functions of the target of rapamycin protein (TOR). Proc Natl Acad Sci U S A 97: 13227–13232. 11078525 10.1073/pnas.240444197 PMC27207

[57] Costanzo M, Baryshnikova A, Bellay J, Kim Y, Spear ED, et al (2010) The genetic landscape of a cell. Science 327: 425–431. 20093466 10.1126/science.1180823 PMC5600254

[58] Ito T, Chiba T, Ozawa R, Yoshida M, Hattori M, et al (2001) A comprehensive two-hybrid analysis to explore the yeast protein interactome. Proc Natl Acad Sci U S A 98: 4569–4574. 11283351 10.1073/pnas.061034498 PMC31875

[59] Flinn RJ, Yan Y, Goswami S, Parker PJ, Backer JM (2010) The late endosome is essential for mTORC1 signaling. Mol Biol Cell 21: 833–841. 20053679 10.1091/mbc.E09-09-0756 PMC2828969

[60] Sturgill TW, Cohen A, Diefenbacher M, Trautwein M, Martin DE, et al (2008) TOR1 and TOR2 have distinct locations in live cells. Eukaryot Cell 7: 1819–1830. 18723607 10.1128/EC.00088-08 PMC2568074

[61] Eldakak A, Rancati G, Rubinstein B, Paul P, Conaway V, et al (2010) Asymmetrically inherited multidrug resistance transporters are recessive determinants in cellular replicative ageing. Nat Cell Biol 12: 799–805. 20657593 10.1038/ncb2085 PMC2917193

[62] Plank M, Wuttke D, van Dam S, Clarke SA, de Magalhaes JP (2012) A meta-analysis of caloric restriction gene expression profiles to infer common signatures and regulatory mechanisms. Mol Biosyst 8: 1339–1349. 22327899 10.1039/c2mb05255e

[63] Anderson RM, Bitterman KJ, Wood JG, Medvedik O, Sinclair DA (2003) Nicotinamide and PNC1 govern lifespan extension by calorie restriction in Saccharomyces cerevisiae. Nature 423: 181–185. 12736687 10.1038/nature01578 PMC4802858

[64] Jaenisch R, Bird A (2003) Epigenetic regulation of gene expression: how the genome integrates intrinsic and environmental signals. Nat Genet 33 Suppl: 245–254. 12610534 10.1038/ng1089

[65] Burdge GC, Hanson MA, Slater-Jefferies JL, Lillycrop KA (2007) Epigenetic regulation of transcription: a mechanism for inducing variations in phenotype (fetal programming) by differences in nutrition during early life? Br J Nutr 97: 1036–1046. 17381976 10.1017/S0007114507682920 PMC2211525

[66] Feser J, Tyler J (2011) Chromatin structure as a mediator of aging. FEBS Lett 585: 2041–2048. 21081125 10.1016/j.febslet.2010.11.016 PMC3988783

[67] Vaquero A, Reinberg D (2009) Calorie restriction and the exercise of chromatin. Genes Dev 23: 1849–1869. 19608767 10.1101/gad.1807009 PMC2725938

[68] Unal E, Kinde B, Amon A (2011) Gametogenesis eliminates age-induced cellular damage and resets life span in yeast. Science 332: 1554–1557. 21700873 10.1126/science.1204349 PMC3923466

[69] Mair W, Goymer P, Pletcher SD, Partridge L (2003) Demography of dietary restriction and death in Drosophila. Science 301: 1731–1733. 14500985 10.1126/science.1086016

[70] Brawley JV, Ferro AJ (1979) Polyamine biosynthesis during germination of yeast ascospores. J Bacteriol 140: 649–654. 387744 10.1128/jb.140.2.649-654.1979 PMC216693

[71] Tabor CW (1981) Mutants of Saccharomyces cerevisiae deficient in polyamine biosynthesis: studies on the regulation of ornithine decarboxylase. Med Biol 59: 272–278. 7040829

[72] Jin Y, Bok JW, Guzman-de-Pena D, Keller NP (2002) Requirement of spermidine for developmental transitions in Aspergillus nidulans. Mol Microbiol 46: 801–812. 12410837 10.1046/j.1365-2958.2002.03201.x

[73] Matsumoto M, Kurihara S, Kibe R, Ashida H, Benno Y (2011) Longevity in mice is promoted by probiotic-induced suppression of colonic senescence dependent on upregulation of gut bacterial polyamine production. PLoS ONE 6: e23652 doi:10.1371/journal.pone.0023652. 21858192 10.1371/journal.pone.0023652 PMC3156754

[74] Soda K, Dobashi Y, Kano Y, Tsujinaka S, Konishi F (2009) Polyamine-rich food decreases age-associated pathology and mortality in aged mice. Exp Gerontol 44: 727–732. 19735716 10.1016/j.exger.2009.08.013

[75] Pnueli L, Edry I, Cohen M, Kassir Y (2004) Glucose and nitrogen regulate the switch from histone deacetylation to acetylation for expression of early meiosis-specific genes in budding yeast. Mol Cell Biol 24: 5197–5208. 15169885 10.1128/MCB.24.12.5197-5208.2004 PMC419861

[76] de Magalhaes JP, Costa J, Toussaint O (2005) HAGR: the Human Ageing Genomic Resources. Nucleic Acids Res 33: D537–543. 15608256 10.1093/nar/gki017 PMC539971

[77] Fischer S, Brunk BP, Chen F, Gao X, Harb OS, et al (2011) Using OrthoMCL to assign proteins to OrthoMCL-DB groups or to cluster proteomes into new ortholog groups. Curr Protoc Bioinformatics Chapter 6 Unit 6 12 11–19. 10.1002/0471250953.bi0612s35 PMC3196566 21901743

[78] Ostlund G, Schmitt T, Forslund K, Kostler T, Messina DN, et al (2010) InParanoid 7: new algorithms and tools for eukaryotic orthology analysis. Nucleic Acids Res 38: D196–203. 19892828 10.1093/nar/gkp931 PMC2808972

[79] Koonin EV, Wolf YI (2010) Constraints and plasticity in genome and molecular-phenome evolution. Nat Rev Genet 11: 487–498. 20548290 10.1038/nrg2810 PMC3273317

[80] Aranda B, Achuthan P, Alam-Faruque Y, Armean I, Bridge A, et al (2010) The IntAct molecular interaction database in 2010. Nucleic Acids Res 38: D525–531. 19850723 10.1093/nar/gkp878 PMC2808934

[81] Xenarios I, Salwinski L, Duan XJ, Higney P, Kim SM, et al (2002) DIP, the Database of Interacting Proteins: a research tool for studying cellular networks of protein interactions. Nucleic Acids Res 30: 303–305. 11752321 10.1093/nar/30.1.303 PMC99070

[82] Ceol A, Chatr Aryamontri A, Licata L, Peluso D, Briganti L, et al (2010) MINT, the molecular interaction database: 2009 update. Nucleic Acids Res 38: D532–539. 19897547 10.1093/nar/gkp983 PMC2808973

[83] Isserlin R, El-Badrawi RA, Bader GD (2011) The Biomolecular Interaction Network Database in PSI-MI 2.5. Database (Oxford) 2011: baq037. 21233089 10.1093/database/baq037 PMC3021793

[84] Breitkreutz BJ, Stark C, Reguly T, Boucher L, Breitkreutz A, et al (2008) The BioGRID Interaction Database: 2008 update. Nucleic Acids Res 36: D637–640. 18000002 10.1093/nar/gkm1001 PMC2238873

[85] Guldener U, Munsterkotter M, Oesterheld M, Pagel P, Ruepp A, et al (2006) MPact: the MIPS protein interaction resource on yeast. Nucleic Acids Res 34: D436–441. 16381906 10.1093/nar/gkj003 PMC1347366

[86] Stein LD (2004) Using the Reactome database. Curr Protoc Bioinformatics Chapter 8 Unit 8 7. 10.1002/0471250953.bi0807s7 18428737

[87] Keshava Prasad TS, Goel R, Kandasamy K, Keerthikumar S, Kumar S, et al (2009) Human Protein Reference Database–2009 update. Nucleic Acids Res 37: D767–772. 18988627 10.1093/nar/gkn892 PMC2686490

[88] Beuming T, Skrabanek L, Niv MY, Mukherjee P, Weinstein H (2005) PDZBase: a protein-protein interaction database for PDZ-domains. Bioinformatics 21: 827–828. 15513994 10.1093/bioinformatics/bti098

[89] Ruepp A, Waegele B, Lechner M, Brauner B, Dunger-Kaltenbach I, et al (2010) CORUM: the comprehensive resource of mammalian protein complexes–2009. Nucleic Acids Res 38: D497–501. 19884131 10.1093/nar/gkp914 PMC2808912

[90] Razick S, Magklaras G, Donaldson IM (2008) iRefIndex: a consolidated protein interaction database with provenance. BMC Bioinformatics 9: 405. 18823568 10.1186/1471-2105-9-405 PMC2573892

[91] Hornbeck PV, Chabra I, Kornhauser JM, Skrzypek E, Zhang B (2004) PhosphoSite: A bioinformatics resource dedicated to physiological protein phosphorylation. Proteomics 4: 1551–1561. 15174125 10.1002/pmic.200300772

[92] Stark C, Su TC, Breitkreutz A, Lourenco P, Dahabieh M, et al (2010) PhosphoGRID: a database of experimentally verified in vivo protein phosphorylation sites from the budding yeast Saccharomyces cerevisiae. Database (Oxford) 2010: bap026. 20428315 10.1093/database/bap026 PMC2860897

[93] Brown KR, Jurisica I (2007) Unequal evolutionary conservation of human protein interactions in interologous networks. Genome Biol 8: R95. 17535438 10.1186/gb-2007-8-5-r95 PMC1929159

[94] Michaut M, Kerrien S, Montecchi-Palazzi L, Chauvat F, Cassier-Chauvat C, et al (2008) InteroPORC: automated inference of highly conserved protein interaction networks. Bioinformatics 24: 1625–1631. 18508856 10.1093/bioinformatics/btn249

[95] Wiles AM, Doderer M, Ruan J, Gu TT, Ravi D, et al (2010) Building and analyzing protein interactome networks by cross-species comparisons. BMC Syst Biol 4: 36. 20353594 10.1186/1752-0509-4-36 PMC2859380

[96] Jayapandian M, Chapman A, Tarcea VG, Yu C, Elkiss A, et al (2007) Michigan Molecular Interactions (MiMI): putting the jigsaw puzzle together. Nucleic Acids Res 35: D566–571. 17130145 10.1093/nar/gkl859 PMC1716720

[97] Tarcea VG, Weymouth T, Ade A, Bookvich A, Gao J, et al (2009) Michigan molecular interactions r2: from interacting proteins to pathways. Nucleic Acids Res 37: D642–646. 18978014 10.1093/nar/gkn722 PMC2686565

[98] Wu J, Vallenius T, Ovaska K, Westermarck J, Makela TP, et al (2009) Integrated network analysis platform for protein-protein interactions. Nat Methods 6: 75–77. 19079255 10.1038/nmeth.1282

[99] Lee I, Li Z, Marcotte EM (2007) An improved, bias-reduced probabilistic functional gene network of baker's yeast, Saccharomyces cerevisiae. PLoS ONE 2: e988 doi:10.1371/journal.pone.0000988. 17912365 10.1371/journal.pone.0000988 PMC1991590

[100] Lee I, Lehner B, Vavouri T, Shin J, Fraser AG, et al (2010) Predicting genetic modifier loci using functional gene networks. Genome Res 20: 1143–1153. 20538624 10.1101/gr.102749.109 PMC2909577

[101] Kim WK, Krumpelman C, Marcotte EM (2008) Inferring mouse gene functions from genomic-scale data using a combined functional network/classification strategy. Genome Biol 9 Suppl 1: S5. 10.1186/gb-2008-9-s1-s5 PMC2447539 18613949

[102] Ravasi T, Suzuki H, Cannistraci CV, Katayama S, Bajic VB, et al (2010) An atlas of combinatorial transcriptional regulation in mouse and man. Cell 140: 744–752. 20211142 10.1016/j.cell.2010.01.044 PMC2836267

[103] Cline MS, Smoot M, Cerami E, Kuchinsky A, Landys N, et al (2007) Integration of biological networks and gene expression data using Cytoscape. Nat Protoc 2: 2366–2382. 17947979 10.1038/nprot.2007.324 PMC3685583

[104] Huang da W, Sherman BT, Tan Q, Kir J, Liu D, et al (2007) DAVID Bioinformatics Resources: expanded annotation database and novel algorithms to better extract biology from large gene lists. Nucleic Acids Res 35: W169–175. 17576678 10.1093/nar/gkm415 PMC1933169

[105] Cherry JM, Hong EL, Amundsen C, Balakrishnan R, Binkley G, et al (2012) Saccharomyces Genome Database: the genomics resource of budding yeast. Nucleic Acids Res 40: D700–705. 22110037 10.1093/nar/gkr1029 PMC3245034

[106] Fisher RA (1932) Statistical methods for research workers. Edinburgh etc.: Oliver and Boyd. xiii p., 1 l. p.

[107] Whitlock MC (2005) Combining probability from independent tests: the weighted Z-method is superior to Fisher's approach. J Evol Biol 18: 1368–1373. 16135132 10.1111/j.1420-9101.2005.00917.x

[108] Wang YX, Catlett NL, Weisman LS (1998) Vac8p, a vacuolar protein with armadillo repeats, functions in both vacuole inheritance and protein targeting from the cytoplasm to vacuole. J Cell Biol 140: 1063–1074. 9490720 10.1083/jcb.140.5.1063 PMC2132703

[109] Gebre S, Connor R, Xia Y, Jawed S, Bush JM, et al (2012) Osh6 overexpression extends the lifespan of yeast by increasing vacuole fusion. Cell Cycle 11: 2176–2188. 22622083 10.4161/cc.20691

[110] Colomina N, Liu Y, Aldea M, Gari E (2003) TOR regulates the subcellular localization of Ime1, a transcriptional activator of meiotic development in budding yeast. Mol Cell Biol 23: 7415–7424. 14517308 10.1128/MCB.23.20.7415-7424.2003 PMC230322

[111] Balderhaar HJ, Arlt H, Ostrowicz C, Brocker C, Sundermann F, et al (2010) The Rab GTPase Ypt7 is linked to retromer-mediated receptor recycling and fusion at the yeast late endosome. J Cell Sci 123: 4085–4094. 21062894 10.1242/jcs.071977

