# Supplementary figures and images for: Dissecting the Gene Network of Dietary Restriction to Identify Evolutionarily Conserved Pathways and New Functional Genes
Source: PLoS Genet. 2012 Aug 9;8(8):e1002834. doi: 10.1371/journal.pgen.1002834 (PMC3415404; doi:10.1371/journal.pgen.1002834)

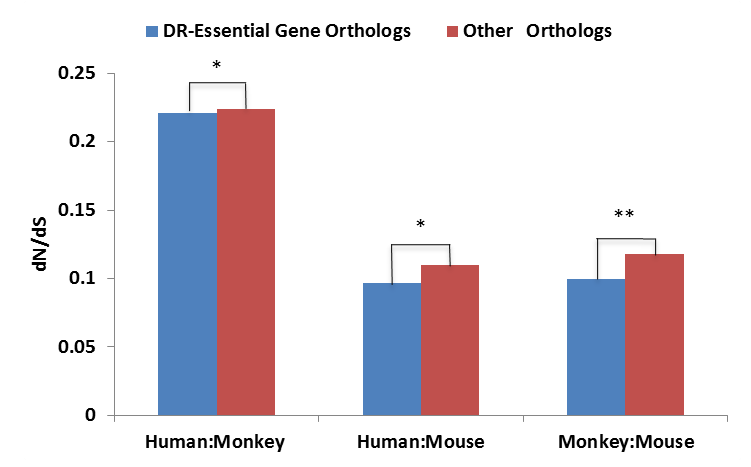

Supplement: Figure S1 — DR-essential genes have low dN/dS ratios. Mammalian DR-essential orthologs have lower dN/dS ratio than expected by chance. (TIF) [file pgen.1002834.s001.tif]

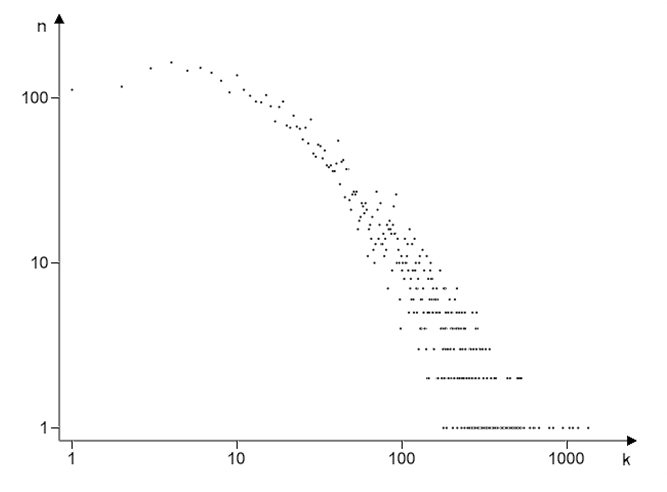

Supplement: Figure S2 — DR-essential gene orthologs in humans form a scale-free network. Degree distribution of human ortholog-complemented DR-essential gene network, as a log-log plot: i.e. log[degree (k)] is plotted against the log of the number of nodes with degree k (n). (TIF) [file pgen.1002834.s002.tif]

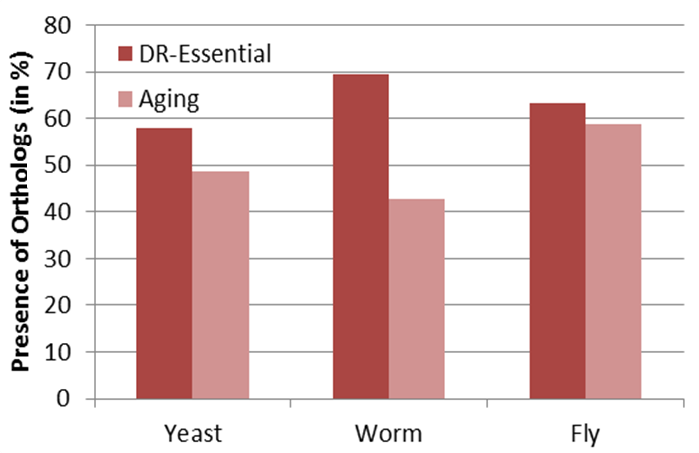

Supplement: Figure S3 — DR-essential genes are more conserved than aging-related genes. DR-essential genes have a higher abundance of orthologs than aging-related genes. (TIF) [file pgen.1002834.s003.tif]

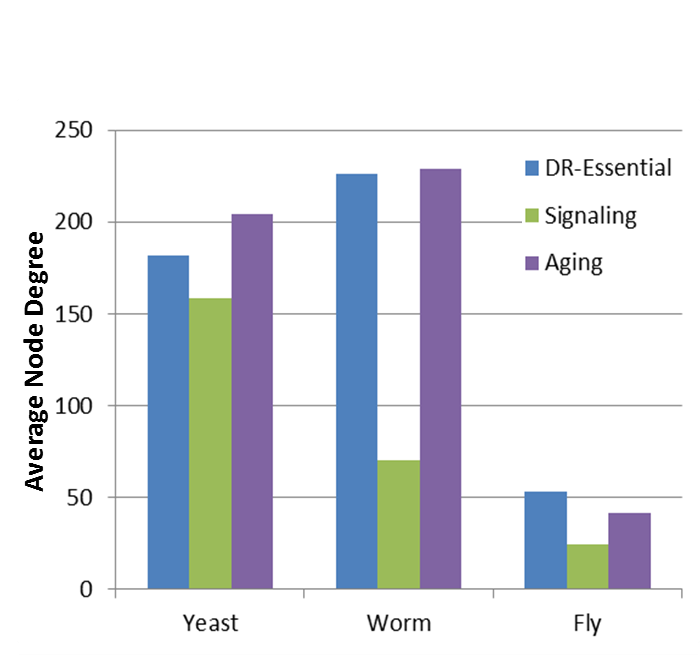

Supplement: Figure S4 — DR-essential genes have a higher node degree than aging-related and signaling genes. DR-essential genes exhibit a high average node degree relative to aging-related genes or signaling genes. (TIF) [file pgen.1002834.s004.tif]
